# UK Medical Cannabis Registry: An Updated Analysis of Inflammatory Arthritis

**DOI:** 10.1177/11795441261480709

**Published:** 2026-09-09

**Authors:** Anusha Sajeev, Simon Erridge, Aritra Datta, Evonne Clarke, Katy McLachlan, Ross Coomber, James J. Rucker, Michael Platt, Mikael H. Sodergren

**Affiliations:** 1 Medical Cannabis Research Group, Imperial College London, UK; 2 Curaleaf Clinic, London, UK; 3 Department of Surgery and Cancer, Imperial College London, UK; 4 St George’s University Hospitals NHS Foundation Trust, London, UK; 5 Department of Psychological Medicine, King’s College London, UK; 6 South London and Maudsley NHS Foundation Trust, UK

**Keywords:** cannabis, tetrahydrocannabinol, cannabidiol, pain, arthritis

## Abstract

**Aim:**

Cannabis-based medicinal products (CBMPs) are an emerging therapeutic option for pain in inflammatory arthritis, yet clinical evidence remains limited. This study aimed to evaluate changes in pain-specific and general health-related outcomes in patients with inflammatory arthritis treated with CBMPs and to characterize the safety profile by examining the incidence and nature of adverse events.

**Methods:**

Patients treated with CBMPs for inflammatory arthritis–associated pain for ⩾24 months were identified from the UK Medical Cannabis Registry. Primary outcomes included changes in patient-reported measures: Brief Pain Inventory (BPI), Pain Visual Analogue Scale (Pain VAS), Short-Form McGill Pain Questionnaire 2 (SF-MPQ-2), EuroQol 5-dimension 5-level (EQ-5D-5L), Generalized Anxiety Disorder-7 (GAD-7), and Single-Item Sleep Quality Scale (SQS) at 1, 3, 6, 12, 18, and 24 months vs baseline. Adverse events were recorded and analysed. Statistical significance was set at *P* < .050.

**Results:**

A total of 192 patients met inclusion criteria. The CBMP initiation was associated with improvements in BPI Interference, BPI Severity, Pain VAS, SF-MPQ-2, SQS, and EQ-5D-5L index values at all follow-up time points (*P* < .010) and in GAD-7 scores up to 3 months (*P* < .001). Twenty-seven patients (14.06%) reported 296 adverse events: 126 (42.57%) mild, 132 (44.59%) moderate, and 38 (12.84%) severe. No life-threatening events occurred.

**Conclusion:**

The CBMP therapy was associated with reductions in pain and improvements in health-related quality of life among individuals with inflammatory arthritis. Although causality cannot be inferred from this observational design, these findings support the need for randomized controlled trials to determine the efficacy of CBMPs for inflammatory arthritis–related pain.

## Introduction

Inflammatory arthritis is an umbrella term for a collection of chronic inflammatory conditions, including rheumatoid arthritis (RA), psoriatic arthritis (PA), and ankylosing spondylitis (AS) and is characterized by joint pain, chronic inflammation, and systemic features.^1^ Current prevalence estimates for inflammatory arthritis vary globally, and while RA is the most common of these conditions, both PA and AS contribute substantially to the disease burden.^2^ The RA alone affects 17.6 million people worldwide at an age-standardized prevalence rate of 209 cases per 100 000, a 14.4% increase since 1990.^3^

The health burden of inflammatory arthritis encompasses not only the sequelae of the condition itself but also the psychological and social impacts the condition has on a patient’s quality of life. The RA, PA, and AS patients are more likely to experience multimorbidity, including an increased risk of cardiovascular death, functional impairment, treatment resistance, and worse clinical outcomes.^4-8^ Individuals with inflammatory arthritis have a higher risk of disability. Patients with AS, eg, have 3-fold higher odds of disability.^9^ The health care costs associated with inflammatory arthritis are multifaceted, consisting of direct medical expenses and indirect productivity losses. The National Audit Office estimates that RA alone incurs costs of around £560 million GBP a year to the National Health Service in the United Kingdom, with the costs of carers, sick leave, and work-related disability pushing the total cost to £3.8 to 4.75 billion GBP each year.^10^

The treatment approach for inflammatory arthritis is multidisciplinary. The primary aim of therapy is to induce and maintain remission through early, aggressive treatment with disease-modifying antirheumatic drugs (DMARDs).^11-14^ Despite best medical therapy, 30% to 70% of individuals with inflammatory arthritis continue to experience acute exacerbations, depending on disease stage and length of follow-up.^15-18^ Moderate-to-severe chronic pain affects 38.4% of all patients with RA.^19^ In addition, 12% of patients in remission continue to experience chronic pain secondary to pre-existing tissue injury or sensitization.^20^ Existing analgesic options are layered on DMARD therapy and include non–steroidal anti-inflammatory drugs (NSAIDs) and opioids.^21^ Naproxen and etoricoxib are commonly prescribed NSAIDs but effectiveness varies within this class of medications.^22,23^ Despite this, 2% to 10% of patients on NSAIDs are forced to discontinue their treatment due to side effects, including gastrointestinal bleeding and acute kidney injury.^24^ Cyclooxygenase-2 (COX-2) selective inhibitors are often prescribed to reduce the risk of these side effects, yet these have been linked to adverse cardiovascular events.^25,26^ Opioids are not currently recommended by present guidelines.^27^ Yet, they are the most prescribed analgesia for inflammatory arthritis.^28^ The evidence supporting the effectiveness of opioids to treat inflammatory arthritis-associated chronic pain is weak.^29,30^ Concurrently, the risk of opioid treatment, including harms related to dependence and opioid use disorder, likely outweighs potential benefits.^25,31^ Given the limitations of current pain management strategies and the persistent burden of symptoms in inflammatory arthropathies, there remains an urgent need to identify novel therapeutics for the effective symptomatic management of pain in this patient population.

The endocannabinoid system (ECS) is involved in the regulation of a variety of functions, from immune system modulation to synaptic neurotransmission, and is composed of G-coupled protein cannabinoid receptors type 1 (CB1) and type 2 (CB2), the respective endogenous ligands, and the enzymes responsible for their synthesis and degradation.^32,33^ The CB1 is predominantly expressed in the hippocampus, basal ganglia, cerebellum, and olfactory bulb, as well as in the peripheral nervous system.^34^ The CB2, conversely, is primarily found in immune cells and some peripheral tissues, including the gastrointestinal tract, reproductive, and cardiovascular systems.^35^ N-arachidonoylethanolamide (anandamide, AEA) is a high-affinity partial agonist of CB1, with weaker activity at CB2, whereas the other endogenous ligand 2-arachidonoylglycerol (2-AG) is a full agonist of both CB receptors.^32,36,37^ The CB receptor activation produces antinociceptive effects through peripheral inhibition of nociceptive transmission, suppression of pain neurotransmitter release in the spinal dorsal horn, and blockade of ascending nociceptive signals in the thalamus.^38^

Cannabis-based medicinal products (CBMPs) are derived from the *Cannabis sativa* plant. The most abundant phytocannabinoids are (−)trans-Δ^9^-tetrahydrocannabinol (THC) and cannabidiol (CBD). The THC is a partial agonist of both CB1 and CB2.^39^ On the contrary, CBD displays weak affinity for these receptors. It is a negative allosteric modulator of CB1, but its primary mechanism of action is suggested to be via direct and indirect inhibition of the catabolism of the endogenous CB1 agonist anandamide by fatty acid amide hydrolase.^40^

The CB1 agonists, such as THC, reduce the perception and intensity of pain in pre-clinical models by reducing ascending nociceptive signals and enhancing the descending inhibitory pathway in the spinal cord.^38^ Binding of THC to the CB1 receptor inhibits adenylyl cyclase, reducing cyclic adenosine monophosphate (cAMP) levels and hindering neuronal excitability by inhibiting neurotransmitter release and thereby blocking transmission of nociceptive signals.^41,42^ Activation of CB2 receptors by THC is also thought to promote an anti-inflammatory state in chronic pain by modulating microglial activity to increase release of anti-inflammatory cytokines and reduce pro-inflammatory cytokines.^43^ The CBD influences pain perception by interacting with a range of other receptors, namely transient receptor potential (TRP) cation channels, 5-hydroxytryptamine (5-HT)_1A_ receptors, and peroxisome proliferator-activated receptor gamma (PPARγ) to modulate the neurotransmission involved in pain signalling.^44^ Specifically, CBD is a weak agonist of transient receptor potential vanilloid channel type 1 (TRPV1), a channel implicated in inflammatory hyperalgesia. The TRPV1 can become desensitized when continually activated, leading to reduced pain and inflammation.^44^

Meta-analyses of randomized controlled trials (RCTs) examining the efficacy of CBMPs for pain management in humans have provided limited support for cannabinoid interventions. Fisher et al^45^ conducted a systematic review of 36 studies that explored the use of cannabinoids, cannabis, and CBMPs to manage pain and identified minimal therapeutic benefit for neuropathic pain in both short- (<7 days) and medium-term (>4 weeks). Similarly, Wang et al^46^ demonstrated only small to very small improvements in pain relief with non-inhaled CBMPs compared to placebo, with their analysis suggesting participants are only 10% more likely to achieve the minimally important difference of 1 point on validated pain scales when prescribed CBMPs compared to placebo. A further meta-analysis found that cannabinoids produced statistically significant reductions in chronic pain and improvements in quality of life; however, the effect sizes for both outcomes fell below the predefined minimal clinically important difference, suggesting that the observed effects were unlikely to be clinically meaningful.^47^ The studies included across all meta-analyses were characterized by methodological limitations, including high or unclear risk of bias and substantial heterogeneity.^45^ Additionally, the duration of follow-up was consistently brief, with no eligible trial following participants beyond 5.5 months.^46^ These limitations collectively underscore the paucity of high-quality evidence regarding the long-term effects of CBMPs on chronic pain management.

Real-world observational studies have aimed to bridge the gap where there has been limited high-quality RCT evidence. In both Australia and the United Kingdom, observational studies have shown that treatment with CBMPs is associated with improvements in pain, sleep, mood, and health-related quality of life (HRQoL) in chronic pain patients across a heterogeneous group of etiologies. The Quality-of-Life Evaluation Study (QUEST) Initiative in Australia reported that such benefits were maintained over 12 months,^48^ while Project Twenty21 in the United Kingdom demonstrated similar gains at 3 months, including reduced opioid use.^49^ Preliminary findings from Project Twenty21 Australia echoed these outcomes across chronic pain, anxiety, post-traumatic stress disorder (PTSD), and multiple sclerosis, although in a smaller cohort with short follow-up.^50^ Longer-term data from a large Australian registry further supported sustained efficacy and safety for up to 2 years.^51^ Despite consistent positive findings, the observational designs, absence of control groups, and high attrition rates limit the strength of the conclusions and highlight the need for RCTs.

Current evidence focusing on the use of CBMPs for inflammatory arthritis has shown promise too, particularly with respect to pain management. A cross-sectional study with 428 participants found that CBD use was associated with an improvement in pain in 83% of participants, and 60.5% of patients reportedly reduced or stopped other medications after they began CBD use.^52^ A previous observational study by the UK Medical Cannabis Registry (UKMCR) investigated clinical outcomes in a cohort of 82 patients with inflammatory arthritis and reported that the initiation of CBMPs was associated with improvements in pain and HRQoL at 1, 3, 6, and 12 months compared with baseline.^53^ However, as follow-up was limited to 12 months, the durability of these benefits and the long-term safety of CBMP remain uncertain. The present study, with an extended follow-up period of 24 months, aims to determine whether the positive effects of CBMP are sustained with prolonged use and to further evaluate its long-term safety profile.

The primary aim of this study was, therefore, to assess changes in both pain-specific and general health-related outcome measures in patients with inflammatory arthritis treated with CBMPs, by evaluating patient-reported outcomes at baseline, 1, 3, 6, 12, 18, and 24 months. The study also aimed to determine whether benefits observed with CBMP use are sustained over a 24-month follow-up. The secondary aim was to characterize the safety profile of CBMPs in this patient population by investigating the incidence and nature of adverse events.

## Materials and Methods

### Study Overview

The UKMCR was used to conduct a case series of patients treated with CBMPs for inflammatory arthritis-associated chronic pain. Patients were required to complete online patient-reported outcome measures (PROMs) at baseline and subsequently at 1, 3, 6, 12, 18, and 24 months.^54^ Data were stored on a bespoke web-based platform, and patients provided written consent before consecutive enrolment. This observational study complied with the Strengthening the Reporting of Observational Studies in Epidemiology (STROBE) guidelines.^55^ The UKMCR has received ethical approval from the Central Bristol Research Ethics Committee (22/SW/0145).

### Setting and Participants

The UKMCR, established in December 2019, is the UK’s first prospective longitudinal registry that collects anonymized data from patients being prescribed CBMPs in the United Kingdom and Crown Dependencies. The registry is managed and privately owned by Curaleaf Clinic. The CBMPs were prescribed per the standards specified by the Medicines and Healthcare Products Regulatory Agency.^56^ The decision to initiate CBMPs was made by an attending-level pain physician, supported by a multidisciplinary team. All CBMPs conformed to Good Manufacturing Practice standards.^57^

Patients who were aged under 18 years, without baseline PROMs, or not enrolled in the UKMCR for a minimum of 24 months prior to data extraction were excluded from analysis. Individuals prescribed CBMPs for primary indications other than inflammatory arthritis-associated chronic pain were also excluded. There were no additional exclusion criteria. Data were extracted on January 6, 2025.

### Data Collection

Baseline questionnaires and consultations with clinicians were used to retrieve patient demographic information, including age, sex, body mass index (BMI), and occupation. Relevant comorbidities were also collected, and the Charlson Comorbidity Index (CCI), a standardized scoring system that predicts 10-year mortality risk based on the presence of 19 specific comorbid conditions, was calculated.^58,59^

Alcohol, tobacco, and cannabis history were also noted. Alcohol consumption was measured in units per week, and lifetime tobacco exposure was in pack-years. Cannabis status was recorded as one of 3 options: no prior cannabis use (cannabis naïve users), ex-users (had previously used cannabis but not using at time of enrolment), and current users (using cannabis at time of enrolment). In ex-users and current users, lifetime cannabis exposure was quantified using gram-years, calculated as *mean cannabis consumption per day (g/d) × years used*.^60^

Concurrent medications taken at baseline were recorded along with dosage per 24 hours and date of commencement. Any changes to medications could be self-recorded by patients via a bespoke online platform or by clinicians during follow-up sessions.^61^ Patients prescribed opiates during the study were identified, and daily oral morphine equivalent (OME) doses (mg/d) were calculated at baseline and each follow-up.

### Cannabis-Based Medicinal Product Prescriptions

All CBMP prescriptions were recorded, including details on route of administration, formulation, cannabis strains, and THC and CBD doses per 24 hours (mg/d).

### Patient-Reported Outcome Measures

Patients electronically completed PROM questionnaires at baseline and at 1, 3, 6, 12, 18, and 24 months, with patients receiving reminders every 72 hours until complete.

#### Brief Pain Inventory

The Brief Pain Inventory-Short Form (BPI) is a 2-part numerical rating scale (NRS) that assesses pain on its intensity (severity subscale) and its interference (interference subscale) in the patient’s life, using 11 items. Scores range from 0 (no pain/no interference) to 10 (pain as bad as you can imagine/complete interference).^62,63^ The minimal clinically important difference (MCID) is defined as ⩾1.^64^

#### Short-Form McGill Pain Questionnaire 2

The Short-Form McGill Pain Questionnaire 2 (SF-MPQ-2) is used to assess the severity and characteristics of neuropathic and non-neuropathic pain. It uses 22 items that assess pain of 4 domains: continuous, intermittent, neuropathic, and affective pain. Each descriptor is scored on a 0 (none) to 10 (worst possible) NRS. The score for each domain and the total score is calculated from the mean of relevant items for each scale.^62^ The MCID is a change of ⩾1.^65^

#### Pain Visual Analogue Scale

The Pain Visual Analogue Scale (Pain VAS) consists of a 10-cm vertical line, with each end anchored to either ‘no pain’ and ‘worst pain’. Participants are required to assess the severity of their pain over the past 24 hours by marking a position on the scale.^66^ The MCID is a change of ⩾1.^67^

#### Generalized Anxiety Disorder-7 Scale

The Generalized Anxiety Disorder-7 scale (GAD-7) is a 7-item tool used to screen for generalized anxiety disorder and assess severity in research and clinical practice. Patients are asked to record symptoms of anxiety experienced over the past 2 weeks, such as trouble relaxing or feeling nervous, on a numeric scale from 0 (not at all) to 3 (nearly every day). A total score, ranging from 0 to 21, is then categorized into minimal (0-4), mild (5-9), moderate (10-14), and severe (15-21) generalized anxiety.^68^ The MCID is an improvement of 4 or more.^69^

#### Single-Item Sleep Quality Scale

The Single-Item Sleep Quality Scale (SQS) asks participants to self-report sleep quality over the past 7 days, on a scale of 0 to 10, considering hours of sleep, frequency of nighttime waking, early morning waking, and how refreshing the sleep was. The score is categorized as either terrible (0), poor (1-3), fair (4-6), good (7-9), or excellent (10) sleep quality.^70^ The MCID is a change of 2.6 or more.^70^

#### EuroQol 5-Dimension 5-Level

The EuroQol 5-dimension 5-level (EQ-5D-5L) is a questionnaire used to self-assess HRQoL in 5 dimensions: ‘mobility’, ‘self-care’, ‘usual activities’, ‘pain/discomfort’, and ‘anxiety/depression’. For each domain, respondents could select from 5 response levels: ‘no problems’, ‘slight problems’, ‘moderate problems’, ‘severe problems’, and ‘unable to/extreme problems’. An index score is then calculated from the resulting health state, with 1 equating to ‘full health’ and a score <0 representing a quality of life ‘worse than death’.^71^ There is no universal MCID available for the EQ-5D-5L index value.

#### Patients’ Global Impression of Change

The Patients’ Global Impression of Change (PGIC) is a 7-point, numerical scale used by patients to rate changes to their pain and overall quality of life following treatment, with 1 reflecting ‘no change or worse’ and 7 being ‘a great deal better/considerable improvement’.^72^ The PGIC is not completed at baseline, as participants are asked to rate changes since starting treatment.

### Adverse Events

Adverse events were either self-recorded by patients onto the online platform at the time they occurred, during completion of PROMs, or by a clinician during routine follow-up consultations. The type of event, its length, and its severity were recorded using the Common Terminology Criteria for Adverse Events version 4.0.^60^

### Missing Data

Multiple imputation using the Multivariate Imputation by Chained Equations (*mice*) package in R was used to impute missing values from PROMs. Five imputed datasets were subsequently pooled for final analysis.

### Statistical Analysis

Descriptive analysis was used to summarize patient demographic data, medication information, and adverse events. Parametric data are presented as mean value ± standard deviation (SD), and non-parametric data are presented as median (interquartile range [IQR]).

Change in PROMs was assessed using repeated-measures analysis of variance (ANOVA). Those PROMs that were statistically significant on initial ANOVA were further explored with a paired *t*-test with the Bonferroni correction to reduce the risk of type 1 error.

Univariable logistic regression analyses were conducted to determine the association of participant- and treatment-specific variables with the MCID for each PROM or any positive change, where an MCID value does not exist. All variables were subsequently entered into a multivariate regression model to determine the adjusted effect of each predictor while controlling for all other variables. Results are reported as odds ratios (ORs) with 95% confidence intervals (CIs).

Univariable and multivariable logistic regression analyses were also conducted to evaluate the association between the same independent variables and the occurrence of adverse events during 24 months of CBMP treatment.

All analyses were conducted in R Studio (version 2025.5.1.513; Posit Software, Boston, Massachusetts), using the R programming language (version 4.5.1; R Core Team, Vienna, Austria). Statistical significance was defined as *P* < .050.

## Results

### Patient Data

**Figure 1.**
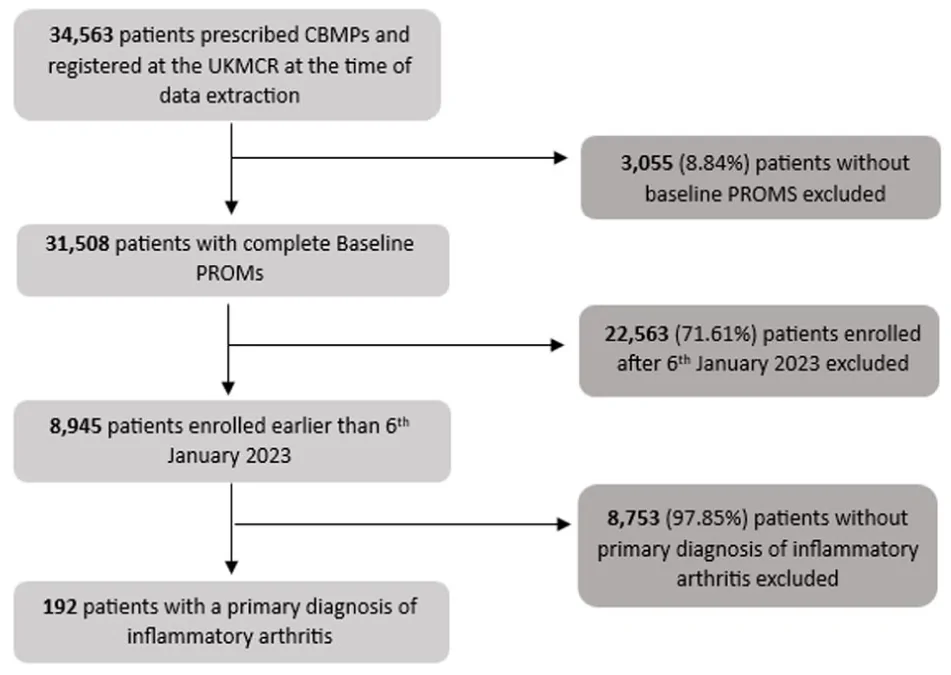
Flowchart of inclusion criteria applied to participants in the UK Medical Cannabis Registry (UKMCR). CBMPs indicates cannabis-based medicinal products; PROMs, patient-reported outcome measures.

A detailed flowchart detailing the application of inclusion criteria to participants in the UKMCR for this study is provided in Figure 1. After applying inclusion criteria, 192 patients remained in the final analysis.

The mean age of the cohort was 46.74 ± 13.43 years (Table 1). There were 103 males (53.65%) included in the analysis. The median BMI was 27.50 (23.20-32.70) kg/m^2^. Most of the cohort was employed (n = 114, 59.38%), whereas 72 (37.50%) were unemployed and 3 (1.56%) patients were retired. At baseline, when treatment with CBMPs was commenced, 69 patients (35.94%) were prescribed opioid medication. Median OME at baseline was 24.00 (6.00-40.00) mg. The median CCI score was 1.00 (0.00-6.00).

**Table 1.** Baseline Patient Demographics.

Demographic details	n (%)/mean ± SD/median [IQR]
Sex	
Male	103 (53.65)
Female	89 (46.35)
Age, years	46.74 ± 13.43
Body mass index, kg/m^2^	27.50 [23.20-32.70]
Occupations	
Unemployed	72 (37.50)
Other occupations	34 (17.71)
Professional	31 (16.15)
Managers	13 (6.77)
Service and sales workers	11 (5.73)
Elementary occupations	7 (3.65)
Clerical support workers	6 (3.13)
Craft and related trades workers	5 (2.60)
Retired	3 (1.56)
Technicians and associate professionals	3 (1.56)
Not reported	3 (1.56)
Plant and machine operators	2 (1.04)
Skilled agricultural, forestry and fishery workers	2 (1.04)
Charlson Comorbidity Index	1.00 [0.00-6.00]
Comorbidities	
Arthritis	192 (100)
Anxiety/depression	56 (29.17)
Hypertension	17 (8.85)
Diabetes	15 (7.81)
Endocrine thyroid dysfunction	12 (6.25)
Venous thromboembolism	5 (2.60)

Abbreviations: IQR, interquartile range; SD, standard deviation.

### Alcohol, Tobacco, and Cannabis Status

The median weekly alcohol consumption among participants was 0.00 (0.00-5.00) units (Table 2). Ex-smoker was the most common smoking status with 87 ex-smokers (45.31%), followed by 63 participants (32.81%) having never smoked before and 42 participants (21.88%) being current smokers. Amongst ex-smokers and current smokers, the median lifetime tobacco exposure was 10 (5.00-20.00) pack-years.

**Table 2.** Cannabis, Tobacco, and Alcohol Status of Participants at Baseline Prior to Initiation of Treatment With Cannabis-Based Medicinal Products.

Cannabis, tobacco, and alcohol status	n (%)/median [IQR]
Cannabis status	
Current user	103 (53.65)
Ex-user	34 (17.71)
Cannabis naïve	55 (28.65)
Frequency of cannabis use (current users)	
Every day	85 (82.52)
Every other day	11 (10.68)
1-2 times per week	6 (5.83)
<1 time per month	1 (0.97)
Cannabis consumption method (current users)	
Vaporizing	79 (76.70)
Smoking	58 (56.31)
Ingestion	42 (40.78)
Topical	8 (7.77)
Current quantity of cannabis consumption g/d (current users)	1.00 [1.00-2.00]
Cannabis use gram-years (current and ex-users)	5.00 [2.00-15.00]
Smoking status	
Current smoker	42 (21.88)
Ex-smoker	87 (45.31)
Never smoked	63 (32.81)
Smoking pack-years (current and ex-smokers)	10.00 [5.00-20.00]
Weekly alcohol consumption in units	0.00 [0.00-5.00]

Abbreviation: IQR, interquartile range.

Most of the cohort were current cannabis users at baseline (n = 103, 53.65%), with the majority using it every day (n = 85, 82.52%). Thirty-four participants (17.71%) were ex-users, and 55 participants (28.65%) had never consumed cannabis before. The median daily quantity of cannabis consumed and the median lifetime of cannabis consumption of current cannabis users were 1.00 (1.00-2.00) g/d and 8.00 (2.75-20.00) gram-years, respectively.

### Cannabis-Based Medicinal Products

Nineteen (9.90%) participants were prescribed dried flower CBMPs only, 79 (41.15%) participants were prescribed oil preparations, and 94 (48.96%) were prescribed both flower and oil CBMPs at baseline (Table 3). The median prescribed CBD dose per day at 24 months was 20.00 [2.00-20.00] mg, while the median THC dose was 20.00 (1.00-22.00) mg per day.

**Table 3.** Proportions of Each Combination of Modality and the (−)Trans-Δ^9^-Tetrahydrocannabinol (THC) and Cannabidiol (CBD) Dose Throughout 24 Months.

CBMP details	Baseline	1 month	3 months	6 months	12 months	18 months	24 months
Cannabinoid doses							
Daily CBD dose (mg/d)	20.00 (2.00-20.00)	20.00 (11.00-20.55)	20.00 (13.67-25.50)	20.49 (16.50-34.41)	20.53 (13.50-46.94)	20.55 (13.38-51.77)	20.54 (12.64-49.50)
Daily THC dose (mg/d)	20.00 (1.00-22.00)	115.5 (7.40-121.00)	120.25 (11.00-168.75)	121.00 (31.63-231.00)	129.25 (32.03-247.50)	131.25 (35.54-260.13)	145.63 (31.75-257.38)
Product combinations							
Oil n (%)	79 (41.15)	65 (33.85)	54 (28.13)	46 (23.96)	47 (24.48)	46 (23.96)	46 (23.96)
Dried flower/flos n (%)	19 (9.90)	18 (9.38)	18 (9.38)	32 (16.67)	41 (21.35)	46 (23.96)	42 (21.88)
Dried flower/flos, oil n (%)	94 (48.96)	108 (56.25)	117 (60.94)	111 (57.81)	102 (53.13)	96 (50.00)	88 (45.83)
Dried flower/flos, pastille n (%)	0 (0.00)	0 (0.00)	0 (0.00)	0 (0.00)	0 (0.00)	0 (0.00)	9 (4.69)
Other n (%)	0 (0.00)	1 (0.52)	3 (1.56)	3 (1.56)	2 (1.04)	4 (2.08)	7 (3.65)

Abbreviation: CBMP, cannabis-based medicinal product.

At 24 months, the most common combination was dried flower/flos and oil (n = 88, 45.83%), followed by oil only (n = 46, 23.96%) and thirdly dried flower/flos only (n = 42, 21.88%). The median prescribed CBD dose per day at 24 months was 20.54 [12.64-49.50] mg, while the median THC dose was 145.63 [31.75-257.38] mg per day.

### Patient-Reported Outcome Measures

Analysis comparing pain-specific PROMs between all time periods showed that there was a difference on the BPI Interference subscale, BPI Severity subscale, Pain VAS, and SF-MPQ-2 total score on repeated-measures ANOVA (*P* < .001).

Improvements in BPI Interference, BPI Severity, Pain VAS, and SF-MPQ-2 were observed between baseline and 1, 3, 6, 12, 18, and 24 months of follow-up (*P* < .001; Table 4).

**Table 4.** Paired Baseline and Follow-up Scores for Health-Related Quality of Life and Pain-Specific Patient-Reported Outcome Measures for Inflammatory Arthritis Patients After 1, 3, 6, 12, 18, and 24 Months of Follow-up.

Patient-reported outcome measures		Baseline	1 month	3 months	6 months	12 months	18 months	24 months
BPI Interference	Score	7.00 ± 2.02	5.17 ± 2.34	4.89 ± 2.63	4.48 ± 3.14	5.61 ± 3.33	5.47 ± 3.79	5.16 ± 3.81
	*P*-value	–	***P* < .001**	***P* < .001**	***P* < .001**	***P* < .001**	***P* < .001**	***P* < .001**
BPI Severity	Score	5.95 ± 1.77	4.79 ± 1.98	4.79 ± 2.00	4.24 ± 2.57	4.59 ± 2.82	4.56 ± 2.92	3.98 ± 2.81
	*P*-value	–	***P* < .001**	***P* < .001**	***P* < .001**	***P* < .001**	***P* < .001**	***P* < .001**
Pain VAS	Score	7.06 ± 1.85	5.53 ± 2.31	5.29 ± 2.66	4.76 ± 2.84	4.91 ± 3.29	4.88 ± 3.71	5.73 ± 3.80
	*P*-value	–	***P* < .001**	***P* < .001**	***P* < .001**	***P* < .001**	***P* < .001**	***P* < .001**
SF-MPQ-2	Score	4.56 ± 1.91	3.52 ± 1.87	3.41 ± 2.00	2.99 ± 1.97	3.46 ± 1.85	3.48 ± 2.78	3.52 ± 3.16
	*P*-value	–	*P* < .001	*P* < .001	*P* < .001	*P* < .001	*P* < .001	*P* = .002
GAD-7	Score	7.42 ± 6.26	4.40 ± 4.35	5.26 ± 5.22	6.28 ± 5.95	6.56 ± 6.88	8.14 ± 7.94	7.38 ± 8.17
	*P*-value	–	***P* < .001**	***P* < .001**	*P* = .505	*P* = 1.000	*P* = 1.000	*P* = 1.000
SQS	Score	3.90 ± 2.28	5.75 ± 2.64	6.16 ± 2.81	6.02 ± 3.12	5.16 ± 3.42	5.32 ± 3.68	5.71 ± 3.87
	*P*-value	–	***P* < .001**	***P* < .001**	***P* < .001**	***P* < .001**	***P* < .001**	***P* < .001**
PGIC	Score	–	5.07 ± 1.55	5.34 ± 1.41	5.11 ± 1.89	5.32 ± 1.75	5.31 ± 2.04	5.28 ± 1.93
	*P*-value	–	–	*P* = .264	*P* = 1.000	*P* = 1.000	*P* = 1.000	*P* = 1.000
EQ-5D-5L mobility	Score	2.98 ± 0.94	2.61 ± 0.98	2.65 ± 1.15	2.47 ± 1.24	2.54 ± 1.24	2.58 ± 1.39	2.57 ± 1.65
	*P*-value	–	***P* < .001**	***P* = .003**	***P* < .001**	***P* < .001**	***P* = .002**	***P* = .025**
EQ-5D-5L self-care	Score	2.32 ± 0.98	2.12 ± 1.02	2.04 ± 0.92	2.01 ± 1.04	2.43 ± 1.26	2.27 ± 1.30	2.42 ± 1.39
	*P*-value	–	*P* = .111	***P* = .002**	***P* = .004**	*P* = 1.000	*P* = 1.000	*P* = 1.000
EQ-5D-5L usual activities	Score	3.20 ± 0.97	2.60 ± 1.04	2.58 ± 1.12	2.70 ± 1.17	2.73 ± 1.13	2.31 ± 1.27	2.88 ± 1.47
	*P*-value	–	*P* < .001	*P* < .001	*P* < .001	*P* < .001	*P* < .001	*P* = .217
EQ-5D-5L pain and discomfort	Score	3.77 ± 0.85	3.01 ± 0.87	2.75 ± 0.95	2.81 ± 1.05	2.65 ± 1.22	2.81 ± 1.21	2.69 ± 1.29
	*P*-value	–	***P* < .001**	***P* < .001**	***P* < .001**	***P* < .001**	***P* < .001**	***P* < .001**
EQ-5D-5L anxiety and depression	Score	2.40 ± 1.13	1.98 ± 0.97	2.02 ± 1.04	1.98 ± 1.06	2.40 ± 1.44	2.44 ± 1.39	2.16 ± 1.32
	*P*-value	–	***P* < .001**	***P* < .001**	***P* < .001**	*P* = 1.000	*P* = 1.000	*P* = .637
EQ-5D-5L index value	Score	0.31 ± 0.28	0.50 ± 0.24	0.49 ± 0.29	0.49 ± 0.30	0.48 ± 0.37	0.47 ± 0.35	0.44 ± 0.45
	*P*-value	–	***P* < .001**	***P* < .001**	***P* < .001**	***P* < .001**	***P* = .002**	***P* = .007**

Scores are presented as mean ± standard deviation. Paired Bonferroni-corrected *P*-values are only calculated for statistically significant *P*-values (*P* < .050) on repeated-measures analysis of variance.

Abbreviations: BPI, Brief Pain Inventory-Short Form; EQ-5D-5L, EuroQol 5-dimension 5-level; GAD-7, Generalized Anxiety Disorder-7; Pain VAS, Pain Visual Analogue Scale; PGIC, Patient Global Impression of Change; SF-MPQ-2, Short-Form McGill Pain Questionnaire 2; SQS, Single-Item Sleep Quality Scale. Values in bold indicate statistically significant differences from baseline (*P *< .050) after Bonferroni correction.

At 24 months, 54.69% (n = 105) of individuals reported clinically significant improvements in BPI Interference, 62.50% (n = 120) in BPI Severity, 49.48% (n = 95) in Pain VAS, and 49.48% (n = 95) in SF-MPQ-2. For HRQoL PROMs at baseline to 1, 3, 6, 12, 18, and 24 months for inflammatory arthritis-associated chronic pain, it showed that for GAD-7, SQS, and EQ-5D-5L index value, there was a difference in mean PROM scores identified on repeated-measures ANOVA (*P* < .001).

Patients reported improvement in GAD-7 between baseline and up to 3 months follow-up (*P* < .001) and in SQS at 1, 3, 6, 12, 18, and 24 months of follow-up compared to baseline (*P* < .001). Overall, the EQ-5D-5L index value displayed an improvement between baseline and all follow-up time periods (*P* < .010).

At 24 months, 32.29% (n = 62) of individuals reported clinically significant improvements in GAD-7 and 45.31% (n = 87) in SQS.

### Adverse Events

A total of 296 adverse events were reported by 27 (14.06%) patients, of which 126 (42.57%) were mild, 132 (44.59%) moderate, and 38 (12.84%) severe (Table 5). There were no life-threatening adverse events reported. The most common adverse events were fatigue, reported 22 times (11.46%), followed by dry mouth (n = 21, 10.94%), insomnia (n = 18, 9.38%), lethargy (n = 17, 8.85%), and somnolence (n = 17, 8.85%).

**Table 5.** Type of Adverse Event and How Many Mild, Moderate, Severe, and Life-Threatening Cases of Each Kind Were Reported.

Adverse event	Count	Percentage (%)	Severity			
			Mild	Moderate	Severe	Life-threatening
Fatigue	22	11.46	3	7	12	0
Dry mouth	21	10.94	16	5	0	0
Insomnia	18	9.38	4	8	6	0
Lethargy	17	8.85	7	10	0	0
Somnolence	17	8.85	0	16	1	0
Dizziness	16	8.33	7	7	2	0
Headache	16	8.33	6	7	3	0
Other (single occurrence)	16	8.33	5	8	3	0
Nausea	15	7.81	12	3	0	0
Concentration impairment	14	7.29	5	9	0	0
Constipation	13	6.77	13	0	0	0
Generalized muscle weakness	11	5.73	4	7	0	0
Cognitive disturbance	9	4.69	2	6	1	0
Abdominal pain	8	4.17	6	2	0	0
Blurred vision	8	4.17	4	3	1	0
Vertigo	8	4.17	4	3	1	0
Anorexia	7	3.65	2	4	1	0
Pharyngitis	7	3.65	0	7	0	0
Amnesia	6	3.13	5	1	0	0
Dysgeusia	6	3.13	4	1	1	0
Ataxia	5	2.60	3	2	0	0
Dyspepsia	5	2.60	2	3	0	0
Tremor	5	2.60	4	1	0	0
Anxiety	4	2.08	0	1	3	0
Confusion	4	2.08	2	1	1	0
Diarrhea	4	2.08	1	3	0	0
Fever	3	1.56	2	1	0	0
Weight loss	3	1.56	3	0	0	0
Lung infection	2	1.04	0	2	0	0
Spasticity	2	1.04	0	1	1	0
Urinary tract infection	2	1.04	0	2	0	0
Weight gain	2	1.04	0	1	1	0
Total	296	154.17	126	132	38	0

All adverse events with only 1 instance have been aggregated into ‘Other’. The total number of adverse events (296) is also displayed.

### Logistic Regression

#### Adverse Events

Univariate analysis showed that treatment with dried flower/flos compared with oil at 24 months was associated with lower odds of adverse events (OR = 0.15, 95% CI = 0.05-0.44, *P* < .001). The full univariate analysis evaluating factors associated with adverse events is presented in Supplementary Table 1.

In the multivariate model, participants with a BMI of 25 to 29.99 kg/m² had higher odds of adverse events compared with those with BMI < 25 kg/m^2^ (OR = 5.88, 95% CI = 1.22-34.95, *P* = .036). In contrast, compared with the lowest THC dose quartile (Min to Q1), both the second quartile (Q1 to median: OR = 0.02, 95% CI = 0.00-0.53, *P* = .043) and the third quartile (median to Q3: OR = 0.01, 95% CI = 0.00-0.34, *P* = .025) were associated with reduced odds of adverse events. Results of the full multivariate logistic regression model for factors associated with adverse events are presented in Supplementary Table 2.

#### Brief Pain Inventory Interference

Univariate analysis indicated that THC dose at 24 months between the median and third quartile (compared with the lowest quartile) was associated with higher odds of an MCID in BPI interference score (OR = 2.39, 95% CI = 1.05-5.59, *P* = .040; Supplementary Table 3).

In the multivariate model, higher CBD dose at 24 months (Q3 to max vs min to Q1) was associated with reduced odds of an MCID in BPI interference score (OR = 0.37, 95% CI = 0.13-0.97, *P* = .047; Supplementary Table 4).

#### Brief Pain Inventory Severity

Univariate analysis showed that baseline SQS scores of 4 to 6 (vs 7-10) were associated with increased odds of clinically significant improvement in BPI Severity (OR = 3.92, 95% CI = 1.55-10.40, *P* = .005). Similarly, baseline SQS scores of 0 to 3 (vs 7-10) were associated with higher odds (OR = 2.71, 95% CI = 1.13-6.80, *P* = .028). In addition, baseline GAD-7 scores of 10 to 14 (vs < 10) were associated with increased odds of reporting an MCID (OR = 3.55, 95% CI = 1.27-12.63, *P* = .026). Detailed univariate analyses of variables associated with BPI Severity score can be found in Supplementary Table 5.

In the multivariate model, these associations remained significant: baseline SQS scores of 4 to 6 (OR = 4.03, 95% CI = 1.37-12.56, *P* = .013) and 0 to 3 (OR = 3.49, 95% CI = 1.24-10.44, *P* = .021) were associated with increased odds of clinically significant improvement in BPI Severity compared with SQS 7-10. Baseline GAD-7 scores of 10 to 14 (vs <10) were also associated with higher odds (OR = 3.59, 95% CI = 1.12-14.30, *P* = .045), while baseline GAD-7 scores ⩾15 (vs <10) were associated with reduced odds (OR = 0.36, 95% CI = 0.13-0.94, *P* = .038). Supplementary Table 6 presents the complete multivariate analysis of factors associated with BPI Severity score.

#### Short-Form McGill Pain Questionnaire 2

Univariate analysis showed that participants aged 51 to 60 years had reduced odds of SF-MPQ-2 clinically significant improvement compared with those aged ⩽30 (OR = 0.26, 95% CI = 0.07-0.89, *P* = .032). Use of dried flower/flos at 24 months (vs oil) was associated with increased odds of improvement (OR = 2.60, 95% CI = 1.11-6.31, *P* = .028). Supplementary Table 7 provides the complete univariate analysis of factors associated with reporting the MCID in SF-MPQ-2.

In the multivariate model, reduced odds of SF-MPQ-2 clinically significant improvements were observed among participants aged 31 to 40 years (OR = 0.22, 95% CI = 0.05-0.83, *P* = .031), 41-50 years (OR = 0.21, 95% CI = 0.05-0.79, *P* = .025), and 51 to 60 years (OR = 0.12, 95% CI = 0.02-0.55, *P* = .008) compared with those aged ⩽30. Additionally, baseline GAD-7 scores ⩾15 (vs <10) were associated with reduced odds of reporting the MCID in SF-MPQ-2 (OR = 0.31, 95% CI = 0.10-0.87, *P* = .031; Supplementary Table 8).

#### Pain Visual Analogue Scale

Univariate analysis showed that THC dose at 24 months was associated with increased odds of an improvement ⩾1 on Pain VAS for participants in the Q1 to median (OR = 3.67, 95% CI = 1.60-8.73, *P* = .003), median to Q3 (OR = 2.39, 95% CI = 1.05-5.59, *P* = .040), and Q3 to max (OR = 2.39, 95% CI = 1.05-5.59, *P* = .040) dose groups compared with the lowest quartile (min to Q1). Use of dried flower/flos (vs oil) was also associated with higher odds of Pain VAS improvement (OR = 4.13, 95% CI = 1.73-10.33, *P* = .002), as was use of other CBMP forms (OR = 4.55, 95% CI = 1.39-16.71, *P* = .015). In addition, baseline GAD-7 scores ⩾15 (vs <10) were associated with increased odds of clinically important improvement (OR = 2.63, 95% CI = 1.21-6.03, *P* = .017; Supplementary Table 9).

In the multivariate model, participants in the Q1 to median THC dose group (vs min to Q1) had significantly higher odds of Pain VAS improvement (OR = 7.17, 95% CI = 1.09-63.53, *P* = .047). Supplementary Table 10 presents the complete multivariate analysis of factors associated with Pain VAS score. Comprehensive outcomes from the univariate and multivariate logistic regression for the odds of reporting an MCID, or an improvement if no MCID is defined, and other PROMs are detailed in Supplementary Tables 11 to 16.

## Discussion

This prospective observational study explored the changes in pain-specific and HRQoL PROMs in patients treated with CBMPs for inflammatory arthritis-associated chronic pain across 24 months. All pain-specific measures showed improvements at each follow-up period. Concurrent improvements were observed in HRQoL as assessed by the EQ-5D-5L and SQS. Symptoms of generalized anxiety improved up to 3 months but showed no change from baseline subsequently. Fewer than 15% of participants reported adverse events throughout the study period, indicating that CBMPs were well-tolerated.

These findings corroborate a previous UKMCR study reporting improvements in pain outcomes up to 12 months in inflammatory arthritis patients treated with CBMPs.^53^ The present study confirms sustained improvement in pain severity and interference up to 2 years using identical validated outcome measures, enabling direct comparison. The QUEST Initiative in Australia similarly reported clinically meaningful improvements in pain among patients prescribed oil-based CBMPs that were maintained over 12 months.^48^ While both studies demonstrated sustained benefits in pain interference and severity throughout follow-up, QUEST utilized the Patient-Reported Outcomes Measurement Information System (PROMIS) rather than the BPI and was restricted to cannabis oil formulations, whereas the present study included dried flower and pastilles.

The Australian Emyria Clinical e-Registry study of almost 4000 participants also demonstrated improvements in pain outcomes among individuals prescribed CBMPs up to 2 years.^51^ The mean differences in BPI Pain Interference and Severity subscales at 24 months in the present study were 1.84 and 1.97, respectively, compared with 1.65 and 1.22 in the Emyria e-Registry. The higher differences in the present study may reflect cohort differences. The present series included individuals consuming cannabis prior to CBMP initiation and was restricted to inflammatory arthritis-associated chronic pain. In addition, Emyria e-Registry participants were prescribed only oil formulations rather than dried flower or pastilles.

At 24 months, 54.7% (n = 105) of participants in the present study reported clinically significant improvements in BPI Interference, 62.5% (n = 120) in BPI Severity, 49.5% (n = 95) in Pain VAS, and 49.5% (n = 95) in SF-MPQ-2. Wang et al^46^ modelled a 10% increased risk difference between CBMPs and placebo for achieving clinically significant improvement in pain severity. Evidence from RCTs estimates that between 35% and 65% of patients with chronic pain report clinically significant improvement in response to placebo.^73-77^ The findings in the present study are largely consistent with the estimated 10% risk difference in reporting clinically significant improvement with CBMPs.^46^ If the lower estimate of clinically significant response to placebo (35%) is accepted, the response in the present study suggests a greater magnitude of effect, which may be multifactorial. Wang et al’s^46^ meta-analysis assessed only oral CBMPs due to a paucity of RCTs on inhaled formulations. Moreover, the placebo effect of CBMPs may be exaggerated due to psychoactive and vasoactive effects, alongside positive media bias.^78,79^

Mean EQ-5D-5L index scores improved from 0.31 ± 0.28 at baseline to 0.44 ± 0.45 at 24 months (*P* < .010). This is consistent with the QUEST Initiative, which reported an adjusted mean improvement of 0.114 ± 0.219 over 12 months in 2353 patients treated with CBMPs.^48^ Both studies showed greatest improvement between baseline and 1 month, which was then maintained throughout follow-up, suggesting initial benefits may be sustained long-term. However, the QUEST study’s broad inclusion criteria encompassing various chronic health conditions may have introduced heterogeneity in treatment response.^48^ Analysis of EQ-5D-5L subscales revealed sustained improvements throughout 24 months only in the mobility and pain/discomfort subscales. This was reflected in a previous 6-month UKMCR study, potentially suggesting that quality of life improvements may be attributable to reductions in pain.^80^ Improvements in the self-care and anxiety/depression subscales were maintained only up to 3 months, indicating short-term benefit that may wane over time due to tolerance or continued disease progression.

In terms of the effect of CBMPs on anxiety, improvements were seen only between baseline and up to 3 months of follow-up, with mean GAD-7 scores instead rising after 3 months up until 24 months of follow-up. Similar outcomes were reported in a previous UKMCR study that compared the effects of medical cannabis in chronic pain patients with and without comorbid anxiety, where median GAD-7 score remained unchanged across follow-up in the no-anxiety group (median = 1.0; IQR = 0.0-3.0), a statistically significant shift in the distribution of scores was observed (*P* < .05), suggesting a subtle overall worsening of anxiety in some participants.^81^ However, it should be highlighted that, as with the present study, the baseline GAD-7 scores in this cohort were already within the mild range, and so these findings may be attributable to scale attenuation, thereby limiting their potential for further improvement. Equally, multivariate logistic regression analyses found that baseline GAD-7 score between 10 and 14 (OR = 9.39, 95% CI = 3.84-24.49, *P* < .001) and 15 and above (OR = 7.59, 95% CI = 3.37-17.74, *P* < .001) were associated with increased odds of positive improvement in GAD-7 score (OR = 9.39, 95% CI = 3.84-24.49, *P* < .001), suggesting that participants with moderate-to-severe anxiety are more likely to experience clinically meaningful benefit from CBMP therapy. Other observational studies did report improvements in generalized anxiety at all lengths of follow-up, but these differences could be attributed to varying baseline GAD-7 scores, CBMP formulations, and shorter follow-up duration.^53,82,83^

Across the 24-month follow-up period, improvements were observed in SQS score at all lengths of follow-up. This is consistent with a previous UKMCR study that explored the effect of CBMP therapy on 1139 chronic pain patients, stratified into sleep-impaired and sleep-unimpaired groups over 12 months.^84^ Improvements in sleep quality from baseline were observed at all time periods up to 12 months in the sleep-impaired cohort.^84^ The magnitude of the improvement was greater than in the sleep-unimpaired cohort, highlighting a differential responsiveness to the CBMP therapy. It should be noted that those in the sleep-impaired arm had worse baseline scores compared to the unimpaired group across all PROMs. The study findings may therefore be representative of the floor and ceiling effects of the SQS, rather than different effects depending on levels of sleep impairment. In comparison between the two studies, there was a reduction in the magnitude of sleep quality improvement in the study including all patients with chronic pain in comparison to the present study. This may be reflective of the development of tolerance to the effects of CBMPs on sleep in the prior study. Conversely, the difference between studies may be better explained by differences in the handling of missing data. The present study uses multiple imputation rather than the baseline observation carried forward method. Consequently, the previous study was biased to reporting a null finding over time with increasing patient attrition, whereas multiple imputation is less prone to such effects. The improvements in sleep quality in the present study align with prior RCTs on CBMPs and sleep, which find improvement in self-reported sleep onset latency, increased sleep time, and sleep efficiency.^85^ Conversely, a study on the effects of cannabinoids on sleep in insomnia, which incorporated high-density electroencephalography, found that although participants reported no next-day impairment, there was no improvement in subjective sleep quality.^86^ Moreover, a single oral dose containing 10 mg THC and 200 mg CBD resulted in suppression of rapid eye movement (REM) sleep.^86^ The present study did not incorporate formal sleep studies and utilized a range of different cannabinoid concentrations across multiple CBMP formats. Future trials should seek to evaluate the effects of a range of different CBMPs on sleep quality and architecture to determine the outcomes across commonly prescribed CBMPs to identify their true effects on sleep quality.

A total of 296 (154.17%) adverse events were reported by 27 patients, an incidence greater than the 57.94% reported by a study exploring the patient-reported safety and efficacy of cannabis from medical cannabis patients in Canada.^87^ In contrast to the present study, where patients could freely report any AEs experienced, patients in the aforementioned study were required to select AEs from a pre-set list of 17 potential options. This difference in protocol may result in underreporting of AEs, potentially underestimating the true incidence of adverse effects. Equally, patients were followed up for 6 weeks as opposed to the 24 months of the present study, meaning overall there was more time for AEs to occur and be reported. Interestingly, the proportion of patients experiencing adverse events in the present study (14.06%) was lower than that reported in the Canadian study (20.00%), suggesting that in the present cohort, individuals who experienced adverse events were more likely to report multiple events.^87^ Francis et al^53^ found the incidence of AEs across 12 months in individuals with inflammatory arthritis was 280.49%, suggesting that prolonged CBMP use is not associated with increased risk when prescribed between 1 and 2 years. The most frequent adverse events were fatigue, dry mouth, insomnia, lethargy, and somnolence, which is consistent with the wider literature.^88,89^ Any differences in adverse event profiles between studies may reflect variability in CBMP formulations, CBD and THC dose, and routes of administration.^90,91^

It is important to interpret the findings of this study in the context of its limitations. Primarily, the observational format meant it was not possible to definitively state that improvements seen in the pain and HRQoL PROMs were solely due to the CBMPs and not due to confounders such as use of other pain medications or additional illicit cannabis use. This was further exacerbated by the lack of a placebo control group and blinding, meaning the potential placebo effect of the CBMPs could not be assessed. Moreover, improvements in outcomes could be reflective of regression to the mean. It should also be noted that the PROMs were a potential cause of recall bias due to their subjective and retrospective nature, while patients may have also overstated the benefits of the CBMP treatment due to increased expectancy bias resulting from positive media coverage and social impressions of the effects of CBMPs.^78,79^

Significant selection bias may also have been present, as the data were obtained from a registry containing patients who all received treatment from the same private clinic, meaning only those who could afford the treatment were included. Nevertheless, the most reported profession was unemployed (37.50%), indicating that socioeconomic status may not influence access to CBMPs. At baseline, 53.65% of patients were current cannabis users, whereas in the United Kingdom between March 2023 and 2024, only 6.8% of people aged 16 to 59 years consumed cannabis and among these individuals, 34% consumed cannabis more than once a month, with 10% using it every day, meaning patients included in the study may not be representative of the UK population.^92^

## Conclusion

Overall, results of this study demonstrate reductions in pain severity and improvements in HRQoL for patients receiving CBMP treatment for chronic pain linked to inflammatory arthritis over a period of 24 months of follow-up. The CBMPs were generally well-tolerated, although the risk of AE and individual responses should be considered before commencing CBMP treatment. These results must be considered in the context of the limitations of the study design, which means that causation cannot be determined. However, it lends support for ongoing evaluation of CBMPs and underscores the need for RCTs for this medication class for inflammatory arthritis-associated chronic pain.

## Supplemental Material

sj-docx-1-amd-10.1177_11795441261480709 – Supplemental material for UK Medical Cannabis Registry: An Updated Analysis of Inflammatory Arthritis Supplemental material, sj-docx-1-amd-10.1177_11795441261480709 for UK Medical Cannabis Registry: An Updated Analysis of Inflammatory Arthritis by Anusha Sajeev, Simon Erridge, Aritra Datta, Evonne Clarke, Katy McLachlan, Ross Coomber, James J. Rucker, Michael Platt and Mikael H. Sodergren in Clinical Medicine Insights: Arthritis and Musculoskeletal Disorders
