# Supplementary material for: UK Medical Cannabis Registry: An Updated Analysis of Inflammatory Arthritis
Source: Clin Med Insights Arthritis Musculoskelet Disord. 2026 Sep 9;19:11795441261480709. doi: 10.1177/11795441261480709 (PMC13558946; doi:10.1177/11795441261480709)
Supplement: Supplementary file 1 [file sj-docx-1-amd-10.1177_11795441261480709.docx]

## **Supplementary Tables**

**Supplementary table 1:** Results of univariate logistic regression analyses evaluating factors associated with the incidence of one or more adverse events in 24 months follow up. Variables assessed include gender, age, body mass index (BMI), cannabis status, cannabis-based medicinal products (CBMPs) at 24 months, cannabidiol (CBD) dose at 24 months, (−)-*trans*-Δ^9^-tetrahydrocannabinol (THC) dose at 24 months, baseline Sleep Quality Scale (SQS), and baseline Generalized Anxiety Disorder-7 (GAD-7) scores.

| **Adverse Events** | | | | |
| --- | --- | --- | --- | --- |
| **Demographic** | | **n** | **Univariate** | |
|  |  |  | **OR (95% CI)** | **P-value** |
| **Gender** | Male (Reference) | 103 | 1.00 (Reference) |  |
|  | Female | 89 | 2.20 (0.96-5.25) | 0.066 |
| **Age** | Under 30 (Reference) | 16 | 1.00 (Reference) |  |
|  | 31-40 | 59 | 0.65 (0.12-4.85) | 0.626 |
|  | 41-50 | 53 | 0.89 (0.18-6.56) | 0.897 |
|  | 51-60 | 33 | 2.24 (0.48-16.23) | 0.347 |
|  | Over 60 | 31 | 1.68 (0.33-12.53) | 0.556 |
| **BMI** | Under 20 | 11 | 1.52 (0.21-7.64) | 0.632 |
|  | 20-24.99 (Reference) | 55 | 1.00 (Reference) |  |
|  | 25-29.99 | 51 | 1.28 (0.42-3.92) | 0.663 |
|  | 30-34.99 | 36 | 1.11 (0.30-3.77) | 0.873 |
|  | 35 and over | 34 | 0.91 (0.22-3.30) | 0.893 |
| **Cannabis status** | Never used (Reference) | 53 | 1.00 (Reference) |  |
|  | Current user | 103 | 0.47 (0.19-1.15) | 0.094 |
|  | Ex-user | 34 | 0.35 (0.07-1.20) | 0.123 |
| **CBMPs 24 months Category** | Oil (Reference) | 46 | 1.00 (Reference) |  |
|  | Dried flower/flos, Oil | 88 | **0.15 (0.05-0.44)** | **<0.001** |
|  | Dried flower/flos | 42 | 0.60 (0.21-1.61) | 0.314 |
|  | Other | 16 | 0.17 (0.01-0.97) | 0.101 |
| **CDB 24 months dose** | Min to Q1 (Reference) | 48 | 1.00 (Reference) |  |
|  | Q1 to Median | 48 | 0.50 (0.14-1.59) | 0.253 |
|  | Median to Q3 | 49 | 0.49 (0.14-1.55) | 0.237 |
|  | Q3 to max | 47 | 0.89 (0.30-2.56) | 0.826 |
| **THC 24 months dose** | Min to Q1 (Reference) | 48 | 1.00 (Reference) |  |
|  | Q1 to Median | 48 | 0.16 (0.04-0.54) | 0.007 |
|  | Median to Q3 | 48 | 0.16 (0.04-0.54) | 0.007 |
|  | Q3 to max | 48 | 0.41 (0.14-1.11) | 0.089 |
| **SQS Baseline** | 7-10 (Reference) | 26 | 1.00 (Reference) |  |
|  | 4-6 | 69 | 1.57 (0.36-10.93) | 0.583 |
|  | 0-3 | 97 | 2.55 (0.67-16.80) | 0.232 |
| **GAD-7 Baseline** | Less than 10 (Reference) | 131 | 1.00 (Reference) |  |
|  | 10-14 | 27 | 1.68 (0.56-4.53) | 0.321 |
|  | 15 and over | 34 | 0.37 (0.06-1.36) | 0.195 |

**Supplementary table 2:** Results of multivariate logistic regression evaluating factors associated with the incidence of one or more adverse events in 24 months follow up.Variables assessed include gender, age, body mass index (BMI), cannabis status, cannabis-based medicinal products (CBMPs) at 24 months, cannabidiol (CBD) dose at 24 months, (−)-trans-Δ^9^-tetrahydrocannabinol (THC) dose at 24 months, baseline Sleep Quality Scale (SQS), and baseline Generalized Anxiety Disorder-7 (GAD-7) scores.

| **Adverse Event** | | | | |
| --- | --- | --- | --- | --- |
| **Demographic** | | **n** | **Multivariate** | |
|  |  |  | **OR (95% CI)** | **P-value** |
| **Gender** | Male (Reference) | 100 | 1.00 (Reference) |  |
|  | Female | 87 | 1.27 (0.32-4.82) | 0.729 |
| **Age** | Under 30 (Reference) | 16 | 1.00 (Reference) |  |
|  | 31-40 | 59 | 0.33 (0.04-3.47) | 0.325 |
|  | 41-50 | 52 | 0.70 (0.10-6.51) | 0.73 |
|  | 51-60 | 32 | 2.05 (0.25-21.44) | 0.515 |
|  | Over 60 | 28 | 0.67 (0.07-7.51) | 0.729 |
| **BMI** | Under 20 | 11 | 3.65 (0.35-32.13) | 0.243 |
|  | 20-24.99 (Reference) | 55 | 1.00 (Reference) |  |
|  | 25-29.99 | 51 | **5.88 (1.22-34.95)** | **0.036** |
|  | 30-34.99 | 36 | 1.04 (0.21-4.99) | 0.963 |
|  | 35 and over | 34 | 0.81 (0.12-4.84) | 0.818 |
| **Cannabis status** | Never used (Reference) | 53 | 1.00 (Reference) |  |
|  | Current user | 101 | 0.93 (0.19-4.81) | 0.93 |
|  | Ex-user | 33 | 0.20 (0.02-1.23) | 0.109 |
| **CBMPs 24 months Category** | Oil (Reference) | 43 | 1.00 (Reference) |  |
|  | Dried flower/flos, Oil | 87 | 2.71 (0.07-248.73) | 0.623 |
|  | Dried flower/flos | 41 | 23.89 (0.58-3315.86) | 0.142 |
|  | Other | 16 | 0.37 (0.01-7.04) | 0.528 |
| **CDB 24 months dose** | Min to Q1 (Reference) | 46 | 1.00 (Reference) |  |
|  | Q1 to Median | 46 | 0.40 (0.07-1.87) | 0.257 |
|  | Median to Q3 | 48 | 0.40 (0.08-1.82) | 0.247 |
|  | Q3 to max | 47 | 1.82 (0.42-8.33) | 0.427 |
| **THC 24 months dose** | Min to Q1 (Reference) | 45 | 1.00 (Reference) |  |
|  | Q1 to Median | 47 | **0.02 (0.00-0.53)** | **0.043** |
|  | Median to Q3 | 47 | **0.01 (0.00-0.34)** | **0.025** |
|  | Q3 to max | 48 | 0.05 (0.00-2.38) | 0.17 |
| **SQS Baseline** | 7-10 (Reference) | 26 | 1.00 (Reference) |  |
|  | 4-6 | 68 | 1.60 (0.24-15.56) | 0.645 |
|  | 0-3 | 93 | 4.89 (0.84-45.26) | 0.107 |
| **GAD-7 Baseline** | Less than 10 (Reference) | 129 | 1.00 (Reference) |  |
|  | 10-14 | 27 | 2.20 (0.52-8.87) | 0.27 |
|  | 15 and over | 31 | 0.25 (0.02-1.68) | 0.199 |

**Supplementary table 3:** Results of univariate logistic regression analyses evaluating factors a minimal clinically important difference in the Brief Pain Inventory-Short Form (BPI) Interference at 24 months. Variables assessed include gender, age, body mass index (BMI), cannabis status, cannabis-based medicinal products (CBMPs) at 24 months, cannabidiol (CBD) dose at 24 months, (−)-trans-Δ^9^-tetrahydrocannabinol (THC) dose at 24 months, baseline Sleep Quality Scale (SQS), and baseline Generalized Anxiety Disorder-7 (GAD-7) scores.

| **BPI Interference** | | | | |
| --- | --- | --- | --- | --- |
| **Demographic** | | **n** | **Univariate** | |
|  |  |  | **OR (95% CI)** | **P-value** |
| **Gender** | Male (Reference) | 103 | 1.00 (Reference) |  |
|  | Female | 89 | 0.80 (0.45-1.41) | 0.438 |
| **Age** | Under 30 (Reference) | 16 | 1.00 (Reference) |  |
|  | 31-40 | 59 | 1.41 (0.45-4.33) | 0.551 |
|  | 41-50 | 53 | 1.10 (0.34-3.39) | 0.874 |
|  | 51-60 | 33 | 0.57 (0.17-1.90) | 0.365 |
|  | Over 60 | 31 | 0.56 (0.16-1.89) | 0.354 |
| **BMI** | Under 20 | 11 | 3.23 (0.75-22.54) | 0.156 |
|  | 20-24.99 (Reference) | 55 | 1.00 (Reference) |  |
|  | 25-29.99 | 51 | 0.81 (0.37-1.74) | 0.588 |
|  | 30-34.99 | 36 | 0.90 (0.38-2.11) | 0.805 |
|  | 35 and over | 34 | 0.64 (0.27-1.51) | 0.308 |
| **Cannabis status** | Never used (Reference) | 55 | 1.00 (Reference) |  |
|  | Current user | 103 | 1.73 (0.90-3.38) | 0.103 |
|  | Ex-user | 34 | 2.37 (0.99-5.85) | 0.056 |
| **CBMPs 24 months Category** | Oil (Reference) | 43 | 1.00 (Reference) |  |
|  | Dried flower/flos, Oil | 88 | 1.19 (0.58-2.45) | 0.625 |
|  | Dried flower/flos | 42 | 2.18 (0.93-5.27) | 0.077 |
|  | Other | 16 | 1.40 (0.45-4.54) | 0.562 |
| **CDB 24 months dose** | Min to Q1 (Reference) | 48 | 1.00 (Reference) |  |
|  | Q1 to Median | 48 | 1.00 (0.44-2.29) | 1 |
|  | Median to Q3 | 49 | 0.53 (0.23-1.19) | 0.125 |
|  | Q3 to max | 47 | 0.53 (0.23-1.19) | 0.126 |
| **THC 24 months dose** | Min to Q1 (Reference) | 48 | 1.00 (Reference) |  |
|  | Q1 to Median | 48 | 1.00 (0.45-2.23) | 1 |
|  | Median to Q3 | 48 |  | **0.04** |
|  | Q3 to max | 48 | 1.28 (0.58-2.88) | 0.54 |
| **SQS Baseline** | 7-10 (Reference) | 26 | 1.00 (Reference) |  |
|  | 4-6 | 69 | 1.09 (0.44-2.71) | 0.85 |
|  | 0-3 | 97 | 1.37 (0.57-3.28) | 0.481 |
| **GAD-7 Baseline** | Less than 10 (Reference) | 131 | 1.00 (Reference) |  |
|  | 10-14 | 27 | 0.99 (0.43-2.32) | 0.987 |
|  | 15 and over | 34 | 0.79 (0.37-1.70) | 0.551 |

**Supplementary table 4:** Results of multivariate logistic regression analyses evaluating factors a minimal clinically important difference in the Brief Pain Inventory-Short Form (BPI) Interference at 24 months. Variables assessed include gender, age, body mass index (BMI), cannabis status, cannabis-based medicinal products (CBMPs) at 24 months, cannabidiol (CBD) dose at 24 months, (−)-trans-Δ^9^-tetrahydrocannabinol (THC) dose at 24 months, baseline Sleep Quality Scale (SQS), and baseline Generalized Anxiety Disorder-7 (GAD-7) scores.

| **BPI Interference** | | | | |
| --- | --- | --- | --- | --- |
| **Demographic** | | **n** | **Multivariate** | |
|  |  |  | **OR (95% CI)** | **P-value** |
| **Gender** | Male (Reference) | 100 | 1.00 (Reference) |  |
|  | Female | 87 | 0.95 (0.42-2.15) | 0.895 |
| **Age** | Under 30 (Reference) | 16 | 1.00 (Reference) |  |
|  | 31-40 | 59 | 1.31 (0.36-4.77) | 0.675 |
|  | 41-50 | 52 | 1.31 (0.36-4.83) | 0.684 |
|  | 51-60 | 32 | 0.66 (0.16-2.82) | 0.578 |
|  | Over 60 | 28 | 0.67 (0.15-2.88) | 0.585 |
| **BMI** | Under 20 | 11 | 2.89 (0.57-22.29) | 0.236 |
|  | 20-24.99 (Reference) | 55 | 1.00 (Reference) |  |
|  | 25-29.99 | 51 | 0.73 (0.30-1.75) | 0.477 |
|  | 30-34.99 | 36 | 1.07 (0.42-2.75) | 0.883 |
|  | 35 and over | 34 | 0.60 (0.22-1.59) | 0.306 |
| **Cannabis status** | Never used (Reference) | 53 | 1.00 (Reference) |  |
|  | Current user | 101 | 1.18 (0.46-3.00) | 0.735 |
|  | Ex-user | 33 | 1.61 (0.58-4.57) | 0.361 |
| **CBMPs 24 months Category** | Oil (Reference) | 43 | 1.00 (Reference) |  |
|  | Dried flower/flos, Oil | 87 | 0.46 (0.06-3.50) | 0.445 |
|  | Dried flower/flos | 41 | 0.76 (0.08-6.67) | 0.797 |
|  | Other | 16 | 0.84 (0.11-6.81) | 0.869 |
| **CDB 24 months dose** | Min to Q1 (Reference) | 46 | 1.00 (Reference) |  |
|  | Q1 to Median | 46 | 1.08 (0.39-2.96) | 0.882 |
|  | Median to Q3 | 48 | 0.56 (0.21-1.50) | 0.253 |
|  | Q3 to max | 47 | **0.37 (0.13-0.97)** | **0.047** |
| **THC 24 months dose** | Min to Q1 (Reference) | 45 | 1.00 (Reference) |  |
|  | Q1 to Median | 47 | 1.90 (0.28-12.98) | 0.498 |
|  | Median to Q3 | 47 | 4.18 (0.54-33.95) | 0.166 |
|  | Q3 to max | 48 | 2.37 (0.31-18.38) | 0.394 |
| **SQS Baseline** | 7-10 (Reference) | 26 | 1.00 (Reference) |  |
|  | 4-6 | 68 | 1.15 (0.42-3.18) | 0.78 |
|  | 0-3 | 93 | 1.80 (0.67-4.90) | 0.24 |
| **GAD-7 Baseline** | Less than 10 (Reference) | 129 | 1.00 (Reference) |  |
|  | 10-14 | 27 | 0.71 (0.27-1.91) | 0.497 |
|  | 15 and over | 31 | 0.63 (0.24-1.67) | 0.357 |

**Supplementary table 5:** Results of univariate logistic regression analyses evaluating factors a minimal clinically important difference in the Brief Pain Inventory-Short Form (BPI) Severity at 24 months. Variables assessed include gender, age, body mass index (BMI), cannabis status, cannabis-based medicinal products (CBMPs) at 24 months, cannabidiol (CBD) dose at 24 months, (−)-trans-Δ^9^-tetrahydrocannabinol (THC) dose at 24 months, baseline Sleep Quality Scale (SQS), and baseline Generalized Anxiety Disorder-7 (GAD-7) scores.

| **BPI Severity** | | | | |
| --- | --- | --- | --- | --- |
| **Demographic** | | **Univariate** | | |
|  |  | **n** | **OR (95% CI)** | **P-value** |
| **Gender** | Male (Reference) | 103 | 1.00 (Reference) |  |
|  | Female | 89 | 0.79 (0.44-1.42) | 0.433 |
| **Age** | Under 30 (Reference) | 16 | 1.00 (Reference) |  |
|  | 31-40 | 59 | 1.22 (0.34-3.95) | 0.744 |
|  | 41-50 | 53 | 0.51 (0.14-1.61) | 0.265 |
|  | 51-60 | 33 | 0.62 (0.16-2.12) | 0.453 |
|  | Over 60 | 31 | 0.72 (0.19-2.53) | 0.615 |
| **BMI** | Under 20 | 11 | 2.57 (0.59-17.97) | 0.255 |
|  | 20-24.99 (Reference) | 55 | 1.00 (Reference) |  |
|  | 25-29.99 | 51 | 1.25 (0.56-2.83) | 0.588 |
|  | 30-34.99 | 36 | 0.90 (0.38-2.16) | 0.808 |
|  | 35 and over | 34 | 0.57 (0.24-1.36) | 0.206 |
| **Cannabis status** | Never used (Reference) | 55 | 1.00 (Reference) |  |
|  | Current user | 103 | 0.90 (0.45-1.76) | 0.76 |
|  | Ex-user | 34 | 1.05 (0.43-2.60) | 0.919 |
| **CBMPs 24 months Category** | Oil (Reference) | 46 | 1.00 (Reference) |  |
|  | Dried flower/flos, Oil | 88 | 1.80 (0.86-3.76) | 0.116 |
|  | Dried flower/flos | 42 | 1.36 (0.58-3.23) | 0.474 |
|  | Other | 16 | 1.08 (0.34-3.49) | 0.895 |
| **CDB 24 months dose** | Min to Q1 (Reference) | 48 | 1.00 (Reference) |  |
|  | Q1 to Median | 48 | 0.76 (0.33-1.75) | 0.525 |
|  | Median to Q3 | 49 | 0.57 (0.25-1.28) | 0.173 |
|  | Q3 to max | 47 | 1.18 (0.50-2.83) | 0.71 |
| **THC 24 months dose** | Min to Q1 (Reference) | 48 | 1.00 (Reference) |  |
|  | Q1 to Median | 48 | 1.54 (0.68-3.54) | 0.3 |
|  | Median to Q3 | 48 | 1.86 (0.81-4.35) | 0.144 |
|  | Q3 to max | 48 | 1.41 (0.63-3.21) | 0.408 |
| **SQS Baseline** | 7-10 (Reference) | 26 | 1.00 (Reference) |  |
|  | 4-6 | 69 | **3.92 (1.55-10.40)** | **0.005** |
|  | 0-3 | 97 | **2.71 (1.13-6.80)** | **0.028** |
| **GAD-7 Baseline** | Less than 10 (Reference) | 131 | 1.00 (Reference) |  |
|  | 10-14 | 27 | **3.55 (1.27-12.63)** | **0.026** |
|  | 15 and over | 34 | 0.55 (0.25-1.17) | 0.122 |

**Supplementary table 6:** Results of multivariate logistic regression analyses evaluating factors a minimal clinically important difference in the Brief Pain Inventory-Short Form (BPI) Severity at 24 months. Variables assessed include gender, age, body mass index (BMI), cannabis status, cannabis-based medicinal products (CBMPs) at 24 months, cannabidiol (CBD) dose at 24 months, (−)-trans-Δ^9^-tetrahydrocannabinol (THC) dose at 24 months, baseline Sleep Quality Scale (SQS), and baseline Generalized Anxiety Disorder-7 (GAD-7) scores.

| **BPI Severity** | | | | |
| --- | --- | --- | --- | --- |
| **Demographic** | | **n** | **Multivariate** | |
|  |  |  | **OR (95% CI)** | **P-value** |
| **Gender** | Male (Reference) | 100 | 1.00 (Reference) |  |
|  | Female | 87 | 0.87 (0.36-2.12) | 0.759 |
| **Age** | Under 30 (Reference) | 16 | 1.00 (Reference) |  |
|  | 31-40 | 59 | 0.66 (0.15-2.58) | 0.563 |
|  | 41-50 | 52 | 0.27 (0.06-1.04) | 0.066 |
|  | 51-60 | 32 | 0.43 (0.09-2.00) | 0.294 |
|  | Over 60 | 28 | 0.47 (0.09-2.20) | 0.348 |
| **BMI** | Under 20 | 11 | 2.28 (0.42-18.52) | 0.377 |
|  | 20-24.99 (Reference) | 55 | 1.00 (Reference) |  |
|  | 25-29.99 | 51 | 1.07 (0.41-2.76) | 0.893 |
|  | 30-34.99 | 36 | 0.59 (0.21-1.63) | 0.309 |
|  | 35 and over | 34 | 0.43 (0.15-1.22) | 0.116 |
| **Cannabis status** | Never used (Reference) | 53 | 1.00 (Reference) |  |
|  | Current user | 101 | 0.62 (0.22-1.74) | 0.373 |
|  | Ex-user | 33 | 1.07 (0.35-3.35) | 0.908 |
| **CBMPs 24 months Category** | Oil (Reference) | 43 | 1.00 (Reference) |  |
|  | Dried flower/flos, Oil | 87 | 0.95 (0.11-8.32) | 0.964 |
|  | Dried flower/flos | 41 | 0.55 (0.05-5.42) | 0.603 |
|  | Other | 16 | 1.13 (0.13-10.87) | 0.909 |
| **CDB 24 months dose** | Min to Q1 (Reference) | 46 | 1.00 (Reference) |  |
|  | Q1 to Median | 46 | 1.13 (0.38-3.36) | 0.831 |
|  | Median to Q3 | 48 | 0.59 (0.21-1.66) | 0.321 |
|  | Q3 to max | 47 | 1.57 (0.54-4.68) | 0.406 |
| **THC 24 months dose** | Min to Q1 (Reference) | 45 | 1.00 (Reference) |  |
|  | Q1 to Median | 47 | 2.38 (0.32-18.57) | 0.386 |
|  | Median to Q3 | 47 | 2.23 (0.26-20.02) | 0.457 |
|  | Q3 to max | 48 | 1.79 (0.21-15.63) | 0.584 |
| **SQS Baseline** | 7-10 (Reference) | 26 | 1.00 (Reference) |  |
|  | 4-6 | 68 | **4.03 (1.37-12.56)** | **0.013** |
|  | 0-3 | 93 | **3.49 (1.24-10.44)** | **0.021** |
| **GAD-7 Baseline** | Less than 10 (Reference) | 129 | 1.00 (Reference) |  |
|  | 10-14 | 27 | **3.59 (1.12-14.30)** | **0.045** |
|  | 15 and over | 31 | **0.36 (0.13-0.94)** | **0.038** |

**Supplementary table 7:** Results of univariate logistic regression analyses evaluating factors a minimal clinically important difference in the Short-Form McGill Pain Questionnaire – Version 2 (SF-MPQ 2). Variables assessed include gender, age, body mass index (BMI), cannabis status, cannabis-based medicinal products (CBMPs) at 24 months, cannabidiol (CBD) dose at 24 months, (−)-trans-Δ^9^-tetrahydrocannabinol (THC) dose at 24 months, baseline Sleep Quality Scale (SQS), and baseline Generalized Anxiety Disorder-7 (GAD-7) scores.

| **SF-MPQ2** | | | | |
| --- | --- | --- | --- | --- |
| **Demographic** | | **n** | **Univariate** | |
|  |  |  | **OR (95% CI)** | **P-value** |
| **Gender** | Male (Reference) | 103 | 1.00 (Reference) |  |
|  | Female | 89 | 0.84 (0.48-1.49) | 0.556 |
| **Age** | Under 30 (Reference) | 16 | 1.00 (Reference) |  |
|  | 31-40 | 59 | 0.54 (0.15-1.68) | 0.302 |
|  | 41-50 | 53 | 0.41 (0.11-1.28) | 0.136 |
|  | **51-60** | 33 | **0.26 (0.07-0.89)** | **0.038** |
|  | Over 60 | 31 | 0.43 (0.11-1.47) | 0.188 |
| **BMI** | Under 20 | 11 | 1.81 (0.49-7.59) | 0.382 |
|  | 20-24.99 (Reference) | 55 | 1.00 (Reference) |  |
|  | 25-29.99 | 51 | 0.85 (0.39-1.83) | 0.681 |
|  | 30-34.99 | 36 | 1.45 (0.62-3.43) | 0.389 |
|  | 35 and over | 34 | 0.73 (0.30-1.71) | 0.467 |
| **Cannabis status** | Never used (Reference) | 53 | 1.00 (Reference) |  |
|  | Current user | 103 | 1.32 (0.68-2.56) | 0.412 |
|  | Ex-user | 34 | 1.64 (0.69-3.92) | 0.263 |
| **CBMPs 24 months Category** | Oil (Reference) | 46 | 1.00 (Reference) |  |
|  | Dried flower/flos, Oil | 88 | 1.03 (0.50-2.14) | 0.926 |
|  | Dried flower/flos | 42 | **2.60 (1.11-6.31)** | **0.031** |
|  | Other | 16 | 1.30 (0.41-4.13) | 0.652 |
| **CDB 24 months dose** | Min to Q1 (Reference) | 48 | 1.00 (Reference) |  |
|  | Q1 to Median | 48 | 1.09 (0.48-2.46) | 0.837 |
|  | Median to Q3 | 49 | 0.45 (0.20-1.01) | 0.056 |
|  | Q3 to max | 47 | 0.68 (0.30-1.53) | 0.358 |
| **THC 24 months dose** | Min to Q1 (Reference) | 48 | 1.00 (Reference) |  |
|  | Q1 to Median | 48 | 1.18 (0.53-2.66) | 0.682 |
|  | Median to Q3 | 48 | 1.65 (0.74-3.74) | 0.222 |
|  | Q3 to max | 48 | 1.29 (0.58-2.89) | 0.54 |
| **SQS Baseline** | 7-10 (Reference) | 26 | 1.00 (Reference) |  |
|  | 4-6 | 69 | 1.75 (0.70-4.50) | 0.236 |
|  | 0-3 | 97 | 1.63 (0.68-4.07) | 0.277 |
| **GAD-7 Baseline** | Less than 10 (Reference) | 131 | 1.00 (Reference) |  |
|  | 10-14 | 27 | 2.03 (0.87-5.05) | 0.111 |
|  | 15 and over | 34 | 0.55 (0.25-1.19) | 0.139 |

**Supplementary table 8:** Results of univariate and multivariate logistic regression analyses evaluating factors a minimal clinically important difference in the Short-Form McGill Pain Questionnaire – Version 2 (SF-MPQ 2). Variables assessed include gender, age, body mass index (BMI), cannabis status, cannabis-based medicinal products (CBMPs) at 24 months, cannabidiol (CBD) dose at 24 months, (−)-trans-Δ^9^-tetrahydrocannabinol (THC) dose at 24 months, baseline Sleep Quality Scale (SQS), and baseline Generalized Anxiety Disorder-7 (GAD-7) scores.

| **SF-MPQ 2** | | | | |
| --- | --- | --- | --- | --- |
| **Demographic** | | **n** | **Multivariate** | |
|  |  |  | **OR (95% CI)** | **P-value** |
| **Gender** | Male (Reference) | 100 | 1.00 (Reference) |  |
|  | Female | 87 | 0.92 (0.40-2.10) | 0.838 |
| **Age** | Under 30 (Reference) | 16 | 1.00 (Reference) |  |
|  | 31-40 | 59 | **0.22 (0.05-0.83)** | **0.031** |
|  | 41-50 | 52 | **0.21 (0.05-0.79)** | **0.025** |
|  | **51-60** | 32 | **0.12 (0.02-0.55)** | **0.008** |
|  | Over 60 | 28 | 0.23 (0.05-1.01) | 0.057 |
| **BMI** | Under 20 | 11 | 1.05 (0.24-5.01) | 0.951 |
|  | 20-24.99 (Reference) | 55 | 1.00 (Reference) |  |
|  | 25-29.99 | 51 | 0.68 (0.28-1.67) | 0.405 |
|  | 30-34.99 | 36 | 1.70 (0.65-4.52) | 0.28 |
|  | 35 and over | 34 | 0.63 (0.22-1.74) | 0.373 |
| **Cannabis status** | Never used (Reference) | 53 | 1.00 (Reference) |  |
|  | Current user | 101 | 0.93 (0.35-2.41) | 0.876 |
|  | Ex-user | 33 | 1.26 (0.44-3.62) | 0.661 |
| **CBMPs 24 months Category** | Oil (Reference) | 43 | 1.00 (Reference) |  |
|  | Dried flower/flos, Oil | 87 | 0.58 (0.07-5.14) | 0.612 |
|  | Dried flower/flos | 41 | 1.67 (0.17-17.05) | 0.654 |
|  | Other | 16 | 1.79 (0.22-15.76) | 0.583 |
| **CDB 24 months dose** | Min to Q1 (Reference) | 46 | 1.00 (Reference) |  |
|  | Q1 to Median | 46 | 1.10 (0.40-3.02) | 0.853 |
|  | Median to Q3 | 48 | 0.42 (0.15-1.13) | 0.088 |
|  | Q3 to max | 47 | 0.45 (0.16-1.22) | 0.118 |
| **THC 24 months dose** | Min to Q1 (Reference) | 45 | 1.00 (Reference) |  |
|  | Q1 to Median | 47 | 2.14 (0.27-16.10) | 0.453 |
|  | Median to Q3 | 47 | 2.70 (0.30-24.67) | 0.37 |
|  | Q3 to max | 48 | 2.43 (0.27-21.38) | 0.416 |
| **SQS Baseline** | 7-10 (Reference) | 26 | 1.00 (Reference) |  |
|  | 4-6 | 68 | 2.34 (0.82-7.06) | 0.118 |
|  | 0-3 | 93 | 2.16 (0.77-6.33) | 0.148 |
| **GAD-7 Baseline** | Less than 10 (Reference) | 129 | 1.00 (Reference) |  |
|  | 10-14 | 27 | 1.89 (0.71-5.31) | 0.209 |
|  | 15 and over | 34 | **0.31 (0.10-0.87)** | **0.031** |

**Supplementary table 9:** Results of univariate logistic regression analyses evaluating factors a minimal clinically important difference in the Visual Analogue Scale (VAS). Variables assessed include gender, age, body mass index (BMI), cannabis status, cannabis-based medicinal products (CBMPs) at 24 months, cannabidiol (CBD) dose at 24 months, (−)-trans-Δ^9^-tetrahydrocannabinol (THC) dose at 24 months, baseline Sleep Quality Scale (SQS), and baseline Generalized Anxiety Disorder-7 (GAD-7) scores.

| **Pain VAS** | | | | |
| --- | --- | --- | --- | --- |
| **Demographic** | | **n** | **Univariate** | |
|  |  |  | **OR (95% CI)** | **P-value** |
| **Gender** | Male (Reference) | 103 | 1.00 (Reference) |  |
|  | Female | 89 | 1.39 (0.79-2.47) | 0.252 |
| **Age** | Under 30 (Reference) | 16 | 1.00 (Reference) |  |
|  | 31-40 | 59 | 0.66 (0.21-1.99) | 0.458 |
|  | 41-50 | 53 | 0.81 (0.25-2.48) | 0.71 |
|  | 51-60 | 33 | 0.73 (0.21-2.43) | 0.611 |
|  | Over 60 | 31 | 0.83 (0.24-2.79) | 0.763 |
| **BMI** | Under 20 | 11 | 1.44 (0.39-5.54) | 0.583 |
|  | 20-24.99 (Reference) | 55 | 1.00 (Reference) |  |
|  | 25-29.99 | 51 | 1.35 (0.63-2.92) | 0.442 |
|  | 30-34.99 | 36 | 0.86 (0.36-2.00) | 0.722 |
|  | 35 and over | 34 | 1.94 (0.82-4.72) | 0.137 |
| **Cannabis status** | Never used (Reference) | 53 | 1.00 (Reference) |  |
|  | Current user | 103 | 0.94 (0.49-1.82) | 0.856 |
|  | Ex-user | 34 | 1.31 (0.56-3.13) | 0.534 |
| **CBMPs 24 months Category** | Oil (Reference) | 46 | 1.00 (Reference) |  |
|  | Dried flower/flos, Oil | 88 | 1.80 (0.87-3.87) | 0.121 |
|  | Dried flower/flos | 42 | **4.13 (1.73-10.33)** | **0.002** |
|  | Other | 16 | **4.55 (1.39-16.71)** | **0.015** |
| **CDB 24 months dose** | Min to Q1 (Reference) | 48 | 1.00 (Reference) |  |
|  | Q1 to Median | 48 | 0.78 (0.35-1.73) | 0.54 |
|  | Median to Q3 | 49 | 0.81 (0.36-1.81) | 0.613 |
|  | Q3 to max | 47 | 1.05 (0.47-2.35) | 0.914 |
| **THC 24 months dose** | Min to Q1 (Reference) | 48 | 1.00 (Reference) |  |
|  | Q1 to Median | 48 | **3.67 (1.60-8.73)** | **0.003** |
|  | Median to Q3 | 48 | **2.39 (1.05-5.59)** | **0.04** |
|  | Q3 to max | 48 | **2.39 (1.05-5.59)** | **0.04** |
| **SQS Baseline** | 7-10 (Reference) | 26 | 1.00 (Reference) |  |
|  | 4-6 | 69 | 2.06 (0.82-5.44) | 0.13 |
|  | 0-3 | 97 | 2.01 (0.83-5.13) | 0.129 |
| **GAD-7 Baseline** | Less than 10 (Reference) | 131 | 1.00 (Reference) |  |
|  | 10-14 | 27 | 1.36 (0.59-3.14) | 0.473 |
|  | 15 and over | 34 | **2.63 (1.21-6.03)** | **0.017** |

**Supplementary table 10:** Results of univariate and multivariate logistic regression analyses evaluating factors a minimal clinically important difference in the Pain Visual Analogue Scale (VAS). Variables assessed include gender, age, body mass index (BMI), cannabis status, cannabis-based medicinal products (CBMPs) at 24 months, cannabidiol (CBD) dose at 24 months, (−)-trans-Δ^9^-tetrahydrocannabinol (THC) dose at 24 months, baseline Sleep Quality Scale (SQS), and baseline Generalized Anxiety Disorder-7 (GAD-7) scores.

| **Pain VAS** | | | | |
| --- | --- | --- | --- | --- |
| **Demographic** | | **n** | **Multivariate** | |
|  |  |  | **OR (95% CI)** | **P-value** |
| **Gender** | Male (Reference) | 100 | 1.00 (Reference) |  |
|  | Female | 87 | 1.29 (0.57-2.94) | 0.541 |
| **Age** | Under 30 (Reference) | 16 | 1.00 (Reference) |  |
|  | 31-40 | 59 | 0.72 (0.19-2.67) | 0.621 |
|  | 41-50 | 52 | 1.05 (0.27-4.02) | 0.943 |
|  | 51-60 | 32 | 0.89 (0.20-3.96) | 0.883 |
|  | Over 60 | 28 | 1.67 (0.38-7.57) | 0.496 |
| **BMI** | Under 20 | 11 | 1.80 (0.41-8.20) | 0.434 |
|  | 20-24.99 (Reference) | 55 | 1.00 (Reference) |  |
|  | 25-29.99 | 51 | 1.51 (0.62-3.77) | 0.365 |
|  | 30-34.99 | 36 | 1.04 (0.39-2.73) | 0.933 |
|  | 35 and over | 34 | 1.87 (0.69-5.23) | 0.223 |
| **Cannabis status** | Never used (Reference) | 53 | 1.00 (Reference) |  |
|  | Current user | 101 | 0.53 (0.19-1.39) | 0.202 |
|  | Ex-user | 33 | 1.04 (0.35-3.08) | 0.942 |
| **CBMPs 24 months Category** | Oil (Reference) | 43 | 1.00 (Reference) |  |
|  | Dried flower/flos, Oil | 87 | 0.56 (0.06-4.27) | 0.584 |
|  | Dried flower/flos | 41 | 1.73 (0.16-15.61) | 0.628 |
|  | Other | 16 | 1.23 (0.12-9.96) | 0.848 |
| **CDB 24 months dose** | Min to Q1 (Reference) | 46 | 1.00 (Reference) |  |
|  | Q1 to Median | 46 | 1.02 (0.37-2.85) | 0.966 |
|  | Median to Q3 | 48 | 0.90 (0.33-2.47) | 0.839 |
|  | Q3 to max | 47 | 0.88 (0.32-2.39) | 0.801 |
| **THC 24 months dose** | Min to Q1 (Reference) | 45 | 1.00 (Reference) |  |
|  | Q1 to Median | 47 | **7.17 (1.09-63.53)** | **0.047** |
|  | Median to Q3 | 47 | 3.75 (0.49-37.49) | 0.216 |
|  | Q3 to max | 48 | 5.55 (0.73-55.75) | 0.11 |
| **SQS Baseline** | 7-10 (Reference) | 26 | 1.00 (Reference) |  |
|  | 4-6 | 68 | 2.72 (0.93-8.46) | 0.073 |
|  | 0-3 | 93 | 1.99 (0.71-5.89) | 0.196 |
| **GAD-7 Baseline** | Less than 10 (Reference) | 129 | 1.00 (Reference) |  |
|  | 10-14 | 27 | 1.22 (0.47-3.18) | 0.683 |
|  | 15 and over | 31 | 2.69 (0.99-7.76) | 0.057 |

**Supplementary table 11:** Results of univariate logistic regression analyses evaluating factors a positive difference in the EuroQol 5-Dimensions, 5-Levels (EQ-5D-5L) index value. Variables assessed include gender, age, body mass index (BMI), cannabis status, cannabis-based medicinal products (CBMPs) at 24 months, cannabidiol (CBD) dose at 24 months, (−)-trans-Δ^9^-tetrahydrocannabinol (THC) dose at 24 months, baseline Sleep Quality Scale (SQS), and baseline Generalized Anxiety Disorder-7 (GAD-7) scores.

| **EQ-5D-5L Index Value** | | | | |
| --- | --- | --- | --- | --- |
| **Univariate** | | **Univariate** | | |
|  |  | **n** | **OR (95% CI)** | **P-value** |
| **Gender** | Male (Reference) | 103 | 1.00 (Reference) |  |
|  | Female | 89 | 1.00 (0.55-1.81) | 0.996 |
| **Age** | Under 30 (Reference) | 16 | 1.00 (Reference) |  |
|  | 31-40 | 59 | 0.94 (0.29-2.89) | 0.914 |
|  | 41-50 | 53 | 1.27 (0.38-4.02) | 0.687 |
|  | 51-60 | 33 | 1.20 (0.33-4.15) | 0.774 |
|  | Over 60 | 31 | 0.95 (0.26-3.27) | 0.936 |
| **BMI** | Under 20 | 11 | 0.86 (0.23-3.32) | 0.824 |
|  | 20-24.99 (Reference) | 55 | 1.00 (Reference) |  |
|  | 25-29.99 | 51 | 1.57 (0.71-3.53) | 0.266 |
|  | 30-34.99 | 36 | 1.63 (0.68-4.07) | 0.279 |
|  | 35 and over | 34 | 1.50 (0.62-3.77) | 0.373 |
| **Cannabis status** | Never used (Reference) | 53 | 1.00 (Reference) |  |
|  | Current user | 103 | 0.62 (0.31-1.24) | 0.183 |
|  | Ex-user | 34 | 1.24 (0.49-3.32) | 0.655 |
| **CBMPs 24 months Category** | Oil (Reference) | 46 | 1.00 (Reference) |  |
|  | Dried flower/flos, Oil | 88 | 0.98 (0.46-2.03) | 0.951 |
|  | Dried flower/flos | 42 | 0.95 (0.40-2.27) | 0.912 |
|  | Other | 16 | 2.54 (0.70-12.23) | 0.189 |
| **CDB 24 months dose** | Min to Q1 (Reference) | 48 | 1.00 (Reference) |  |
|  | Q1 to Median | 48 | 1.10 (0.47-2.61) | 0.827 |
|  | Median to Q3 | 49 | 0.79 (0.34-1.81) | 0.577 |
|  | Q3 to max | 47 | 0.74 (0.32-1.70) | 0.474 |
| **THC 24 months dose** | Min to Q1 (Reference) | 48 | 1.00 (Reference) |  |
|  | Q1 to Median | 48 | 1.46 (0.62-3.46) | 0.387 |
|  | Median to Q3 | 48 | 1.32 (0.57-3.10) | 0.52 |
|  | Q3 to max | 48 | 0.71 (0.31-1.60) | 0.408 |
| **SQS Baseline** | 7-10 (Reference) | 26 | 1.00 (Reference) |  |
|  | 4-6 | 69 | 1.42 (0.56-3.54) | 0.453 |
|  | 0-3 | 97 | 1.82 (0.75-4.42) | 0.181 |
| **GAD-7 Baseline** | Less than 10 (Reference) | 131 | 1.00 (Reference) |  |
|  | 10-14 | 27 | 1.94 (0.80-5.24) | 0.162 |
|  | 15 and over | 34 | 1.89 (0.84-4.57) | 0.137 |

**Supplementary table 12:** Results of multivariate logistic regression analyses evaluating factors a positive difference in the EuroQol 5-Dimensions, 5-Levels (EQ-5D-5L) index value. Variables assessed include gender, age, body mass index (BMI), cannabis status, cannabis-based medicinal products (CBMPs) at 24 months, cannabidiol (CBD) dose at 24 months, (−)-trans-Δ^9^-tetrahydrocannabinol (THC) dose at 24 months, baseline Sleep Quality Scale (SQS), and baseline Generalized Anxiety Disorder-7 (GAD-7) scores.

| **EQ-5D-5L Index Value** | | | | |
| --- | --- | --- | --- | --- |
| **Demographic** | | **n** | **Multivariate** | |
|  |  |  | **OR (95% CI)** | **P-value** |
| **Gender** | Male (Reference) | 100 | 1.00 (Reference) |  |
|  | Female | 87 | 0.66 (0.29-1.49) | 0.315 |
| **Age** | Under 30 (Reference) | 16 | 1.00 (Reference) |  |
|  | 31-40 | 59 | 0.80 (0.21-2.88) | 0.73 |
|  | 41-50 | 52 | 1.30 (0.33-4.89) | 0.699 |
|  | 51-60 | 32 | 0.87 (0.19-3.80) | 0.85 |
|  | Over 60 | 28 | 0.91 (0.20-3.99) | 0.904 |
| **BMI** | Under 20 | 11 | 0.97 (0.23-4.19) | 0.963 |
|  | 20-24.99 (Reference) | 55 | 1.00 (Reference) |  |
|  | 25-29.99 | 51 | 1.54 (0.63-3.82) | 0.342 |
|  | 30-34.99 | 36 | 1.64 (0.62-4.41) | 0.321 |
|  | 35 and over | 34 | 1.20 (0.44-3.32) | 0.721 |
| **Cannabis status** | Never used (Reference) | 53 | 1.00 (Reference) |  |
|  | Current user | 101 | 0.44 (0.16-1.14) | 0.098 |
|  | Ex-user | 33 | 1.01 (0.34-3.17) | 0.979 |
| **CBMPs 24 months Category** | Oil (Reference) | 43 | 1.00 (Reference) |  |
|  | Dried flower/flos, Oil | 87 | 0.33 (0.03-2.90) | 0.32 |
|  | Dried flower/flos | 41 | 0.37 (0.03-3.75) | 0.404 |
|  | Other | 16 | 1.23 (0.14-12.57) | 0.851 |
| **CDB 24 months dose** | Min to Q1 (Reference) | 46 | 1.00 (Reference) |  |
|  | Q1 to Median | 46 | 1.05 (0.37-2.97) | 0.924 |
|  | Median to Q3 | 48 | 0.81 (0.30-2.20) | 0.684 |
|  | Q3 to max | 47 | 0.66 (0.24-1.77) | 0.407 |
| **THC 24 months dose** | Min to Q1 (Reference) | 45 | 1.00 (Reference) |  |
|  | Q1 to Median | 47 | 3.93 (0.52-35.09) | 0.188 |
|  | Median to Q3 | 47 | 4.12 (0.48-40.43) | 0.198 |
|  | Q3 to max | 48 | 2.67 (0.31-25.54) | 0.369 |
| **SQS Baseline** | 7-10 (Reference) | 26 | 1.00 (Reference) |  |
|  | 4-6 | 68 | 1.25 (0.44-3.51) | 0.668 |
|  | 0-3 | 93 | 1.30 (0.47-3.52) | 0.608 |
| **GAD-7 Baseline** | Less than 10 (Reference) | 129 | 1.00 (Reference) |  |
|  | 10-14 | 27 | 2.10 (0.77-6.32) | 0.161 |
|  | 15 and over | 31 | 2.17 (0.79-6.52) | 0.146 |

**Supplementary table 13:** Results of univariate logistic regression analyses evaluating factors a minimal clinically important difference in the Single-Item Sleep Quality Scale (SQS). Variables assessed include gender, age, body mass index (BMI), cannabis status, cannabis-based medicinal products (CBMPs) at 24 months, cannabidiol (CBD) dose at 24 months, (−)-trans-Δ^9^-tetrahydrocannabinol (THC) dose at 24 months, baseline Sleep Quality Scale (SQS), and baseline Generalized Anxiety Disorder-7 (GAD-7) scores.

| **SQS** | | | | |
| --- | --- | --- | --- | --- |
| **Demographic** | | **n** | **Univariate** | |
|  |  |  | **OR (95% CI)** | **P-value** |
| **Gender** | Male (Reference) | 103 | 1.00 (Reference) |  |
|  | Female | 89 | 1.25 (0.71-2.22) | 0.438 |
| **Age** | Under 30 (Reference) | 16 | 1.00 (Reference) |  |
|  | 31-40 | 59 | 1.86 (0.60-6.50) | 0.302 |
|  | 41-50 | 53 | 2.28 (0.72-8.09) | 0.172 |
|  | 51-60 | 33 | 1.43 (0.41-5.42) | 0.58 |
|  | Over 60 | 31 | 2.06 (0.60-7.86) | 0.264 |
| **BMI** | Under 20 | 11 | 0.86 (0.23-3.20) | 0.826 |
|  | 20-24.99 (Reference) | 55 | 1.00 (Reference) |  |
|  | 25-29.99 | 51 | 0.85 (0.39-1.83) | 0.681 |
|  | 30-34.99 | 36 | 0.93 (0.40-2.16) | 0.862 |
|  | 35 and over | 34 | 0.64 (0.26-1.52) | 0.318 |
| **Cannabis status** | Never used (Reference) | 53 | 1.00 (Reference) |  |
|  | Current user | 103 | **0.42 (0.21-0.82)** | **0.011** |
|  | Ex-user | 34 | 0.72 (0.30-1.70) | 0.451 |
| **CBMPs 24 months Category** | Oil (Reference) | 46 | 1.00 (Reference) |  |
|  | Dried flower/flos, Oil | 88 | 0.73 (0.36-1.50) | 0.394 |
|  | Dried flower/flos | 42 | 0.47 (0.19-1.09) | 0.081 |
|  | Other | 16 | 0.50 (0.15-1.59) | 0.25 |
| **CDB 24 months dose** | Min to Q1 (Reference) | 48 | 1.00 (Reference) |  |
|  | Q1 to Median | 48 | 0.85 (0.38-1.89) | 0.683 |
|  | Median to Q3 | 49 | 0.88 (0.40-1.96) | 0.763 |
|  | Q3 to max | 47 | 0.62 (0.27-1.40) | 0.252 |
| **THC 24 months dose** | Min to Q1 (Reference) | 48 | 1.00 (Reference) |  |
|  | Q1 to Median | 48 | 0.47 (0.20-1.05) | 0.068 |
|  | Median to Q3 | 48 | 0.71 (0.32-1.60) | 0.413 |
|  | Q3 to max | 48 | **0.36 (0.15-0.81)** | **0.015** |
| **SQS Baseline** | 7-10 (Reference) | 26 | 1.00 (Reference) |  |
|  | 4-6 | 69 | **5.90 (1.83-26.52)** | **0.007** |
|  | 0-3 | 97 | **9.63 (3.09-42.51)** | **<0.001** |
| **GAD-7 Baseline** | Less than 10 (Reference) | 131 | 1.00 (Reference) |  |
|  | 10-14 | 27 | 0.74 (0.31-1.71) | 0.487 |
|  | 15 and over | 34 | 0.67 (0.30-1.43) | 0.306 |

**Supplementary table 14:** Results of multivariate logistic regression analyses evaluating factors a minimal clinically important difference in the Single-Item Sleep Quality Scale (SQS). Variables assessed include gender, age, body mass index (BMI), cannabis status, cannabis-based medicinal products (CBMPs) at 24 months, cannabidiol (CBD) dose at 24 months, (−)-trans-Δ^9^-tetrahydrocannabinol (THC) dose at 24 months, baseline Sleep Quality Scale (SQS), and baseline Generalized Anxiety Disorder-7 (GAD-7) scores.

| **SQS** | | | | |
| --- | --- | --- | --- | --- |
| **Demographic** | | **n** | **Multivariate** | |
|  |  |  | **OR (95% CI)** | **P-value** |
| **Gender** | Male (Reference) | 100 | 1.00 (Reference) |  |
|  | Female | 87 | 0.91 (0.38-2.13) | 0.82 |
| **Age** | Under 30 (Reference) | 16 | 1.00 (Reference) |  |
|  | 31-40 | 59 | 1.40 (0.36-5.84) | 0.636 |
|  | 41-50 | 52 | 1.86 (0.46-8.09) | 0.389 |
|  | 51-60 | 32 | 0.84 (0.18-4.07) | 0.83 |
|  | Over 60 | 28 | 1.47 (0.31-7.19) | 0.628 |
| **BMI** | Under 20 | 11 | 0.97 (0.21-4.44) | 0.971 |
|  | 20-24.99 (Reference) | 55 | 1.00 (Reference) |  |
|  | 25-29.99 | 51 | 0.73 (0.29-1.84) | 0.511 |
|  | 30-34.99 | 36 | 0.74 (0.27-1.98) | 0.549 |
|  | 35 and over | 34 | 0.58 (0.19-1.70) | 0.324 |
| **Cannabis status** | Never used (Reference) | 53 | 1.00 (Reference) |  |
|  | Current user | 101 | **0.34 (0.13-0.90)** | **0.033** |
|  | Ex-user | 33 | 0.51 (0.18-1.47) | 0.218 |
| **CBMPs 24 months Category** | Oil (Reference) | 43 | 1.00 (Reference) |  |
|  | Dried flower/flos, Oil | 87 | 4.14 (0.46-93.70) | 0.255 |
|  | Dried flower/flos | 41 | 2.60 (0.24-64.04) | 0.471 |
|  | Other | 16 | 2.73 (0.29-65.04) | 0.431 |
| **CDB 24 months dose** | Min to Q1 (Reference) | 46 | 1.00 (Reference) |  |
|  | Q1 to Median | 46 | 0.67 (0.23-1.89) | 0.453 |
|  | Median to Q3 | 48 | 0.79 (0.28-2.21) | 0.653 |
|  | Q3 to max | 47 | 0.42 (0.15-1.15) | 0.096 |
| **THC 24 months dose** | Min to Q1 (Reference) | 45 | 1.00 (Reference) |  |
|  | Q1 to Median | 47 | 0.18 (0.01-1.42) | 0.151 |
|  | Median to Q3 | 47 | 0.36 (0.02-3.24) | 0.409 |
|  | Q3 to max | 48 | 0.20 (0.01-1.82) | 0.201 |
| **SQS Baseline** | 7-10 (Reference) | 26 | 1.00 (Reference) |  |
|  | 4-6 | 68 | **5.05 (1.43-24.16)** | **0.021** |
|  | 0-3 | 93 | **12.38 (3.57-58.96)** | **<0.001** |
| **GAD-7 Baseline** | Less than 10 (Reference) | 129 | 1.00 (Reference) |  |
|  | 10-14 | 27 | 0.44 (0.16-1.16) | 0.103 |
|  | 15 and over | 31 | 0.40 (0.14-1.08) | 0.075 |

**Supplementary table 15:** Results of univariate logistic regression analyses evaluating factors a minimal clinically important difference in the Generalized Anxiety Disorder-7 (GAD-7). Variables assessed include gender, age, body mass index (BMI), cannabis status, cannabis-based medicinal products (CBMPs) at 24 months, cannabidiol (CBD) dose at 24 months, (−)-trans-Δ^9^-tetrahydrocannabinol (THC) dose at 24 months, baseline Sleep Quality Scale (SQS), and baseline Generalized Anxiety Disorder-7 (GAD-7) scores.

| **GAD-7** | | | | |
| --- | --- | --- | --- | --- |
| **Demographic** | | **n** | **Univariate** | |
|  |  |  | **OR (95% CI)** | **P-value** |
| **Gender** | Male (Reference) | 100 | 1.00 (Reference) |  |
|  | **Female** | 89 | **2.01 (1.09-3.74)** | **0.026** |
| **Age** | Under 30 (Reference) | 16 | 1.00 (Reference) |  |
|  | 31-40 | 59 | 0.56 (0.18-1.80) | 0.322 |
|  | 41-50 | 53 | 0.46 (0.14-1.51) | 0.192 |
|  | 51-60 | 33 | 0.48 (0.14-1.70) | 0.253 |
|  | Over 60 | 31 | 1.06 (0.31-3.66) | 0.927 |
| **BMI** | Under 20 | 11 | 2.03 (0.52-7.72) | 0.293 |
|  | 20-24.99 (Reference) | 55 | 1.00 (Reference) |  |
|  | 25-29.99 | 51 | 1.11 (0.48-2.57) | 0.798 |
|  | 30-34.99 | 36 | 1.38 (0.56-3.38) | 0.483 |
|  | 35 and over | 34 | 1.02 (0.39-2.58) | 0.974 |
| **Cannabis status** | Never used (Reference) | 53 | 1.00 (Reference) |  |
|  | Current user | 103 | 0.70 (0.35-1.39) | 0.304 |
|  | Ex-user | 34 | 0.67 (0.26-1.66) | 0.4 |
| **CBMPs 24 months Category** | Oil (Reference) | 43 | 1.00 (Reference) |  |
|  | Dried flower/flos, Oil | 88 | 1.38 (0.64-3.07) | 0.416 |
|  | Dried flower/flos | 42 | 1.14 (0.45-2.86) | 0.782 |
|  | Other | 16 | 1.15 (0.31-3.88) | 0.821 |
| **CDB 24 months dose** | Min to Q1 (Reference) | 46 | 1.00 (Reference) |  |
|  | Q1 to Median | 48 | 1.00 (0.44-2.27) | 1 |
|  | Median to Q3 | 49 | 0.55 (0.23-1.29) | 0.174 |
|  | Q3 to max | 47 | 0.47 (0.19-1.12) | 0.093 |
| **THC 24 months dose** | Min to Q1 (Reference) | 45 | 1.00 (Reference) |  |
|  | Q1 to Median | 48 | 1.10 (0.46-2.66) | 0.824 |
|  | Median to Q3 | 48 | 1.21 (0.51-2.91) | 0.66 |
|  | Q3 to max | 48 | 1.33 (0.57-3.18) | 0.513 |
| **SQS Baseline** | 7-10 (Reference) | 26 | 1.00 (Reference) |  |
|  | 4-6 | 69 | 0.82 (0.30-2.41) | 0.705 |
|  | 0-3 | 97 | 1.83 (0.73-5.05) | 0.218 |
| **GAD-7 Baseline** | Less than 10 (Reference) | 129 | 1.00 (Reference) |  |
|  | 10-14 | 27 | **9.39 (3.84-24.49)** | **<0.001** |
|  | 15 and over | 34 | **7.59 (3.37-17.74)** | **<0.001** |

**Supplementary table 16:** Results of multivariate logistic regression analyses evaluating factors a minimal clinically important difference in the Generalized Anxiety Disorder-7 (GAD-7). Variables assessed include gender, age, body mass index (BMI), cannabis status, cannabis-based medicinal products (CBMPs) at 24 months, cannabidiol (CBD) dose at 24 months, (−)-trans-Δ^9^-tetrahydrocannabinol (THC) dose at 24 months, baseline Sleep Quality Scale (SQS), and baseline Generalized Anxiety Disorder-7 (GAD-7) scores.

| **GAD-7** | | | | |
| --- | --- | --- | --- | --- |
| **Demographic** | | **n** | **Multivariate** | |
|  |  |  | **OR (95% CI)** | **P-value** |
| **Gender** | Male (Reference) | 100 | 1.00 (Reference) |  |
|  | **Female** | 89 | 2.47 (0.97-6.45) | 0.059 |
| **Age** | Under 30 (Reference) | 16 | 1.00 (Reference) |  |
|  | 31-40 | 59 | 0.49 (0.12-2.13) | 0.331 |
|  | 41-50 | 53 | 0.44 (0.10-1.99) | 0.279 |
|  | 51-60 | 33 | 0.36 (0.06-2.07) | 0.247 |
|  | Over 60 | 31 | 1.11 (0.22-6.07) | 0.901 |
| **BMI** | Under 20 | 11 | 1.47 (0.27-7.82) | 0.647 |
|  | 20-24.99 (Reference) | 55 | 1.00 (Reference) |  |
|  | 25-29.99 | 51 | 2.22 (0.77-6.79) | 0.15 |
|  | 30-34.99 | 36 | 1.99 (0.62-6.49) | 0.249 |
|  | 35 and over | 34 | 0.92 (0.26-3.17) | 0.896 |
| **Cannabis status** | Never used (Reference) | 53 | 1.00 (Reference) |  |
|  | Current user | 103 | 0.45 (0.14-1.44) | 0.181 |
|  | Ex-user | 34 | 0.58 (0.16-1.96) | 0.387 |
| **CBMPs 24 months Category** | Oil (Reference) | 43 | 1.00 (Reference) |  |
|  | Dried flower/flos, Oil | 88 | 2.21 (0.20-29.44) | 0.527 |
|  | Dried flower/flos | 42 | 1.46 (0.10-24.37) | 0.785 |
|  | Other | 16 | 0.94 (0.07-12.93) | 0.964 |
| **CDB 24 months dose** | Min to Q1 (Reference) | 46 | 1.00 (Reference) |  |
|  | Q1 to Median | 48 | 0.81 (0.25-2.59) | 0.717 |
|  | Median to Q3 | 49 | 0.39 (0.11-1.28) | 0.127 |
|  | Q3 to max | 47 | 0.27 (0.08-0.87) | 0.033 |
| **THC 24 months dose** | Min to Q1 (Reference) | 45 | 1.00 (Reference) |  |
|  | Q1 to Median | 48 | 0.96 (0.08-9.41) | 0.971 |
|  | Median to Q3 | 48 | 1.33 (0.10-16.02) | 0.82 |
|  | Q3 to max | 48 | 2.94 (0.24-35.66) | 0.387 |
| **SQS Baseline** | 7-10 (Reference) | 26 | 1.00 (Reference) |  |
|  | 4-6 | 69 | 0.50 (0.13-1.95) | 0.305 |
|  | 0-3 | 97 | 1.04 (0.30-3.76) | 0.955 |
| **GAD-7 Baseline** | Less than 10 (Reference) | 129 | 1.00 (Reference) |  |
|  | 10-14 | 27 | **9.03 (3.18-27.89)** | **<0.001** |
|  | 15 and over | 34 | **6.45 (2.29-19.48)** | **<0.001** |
